# Abilities of the BRICHOS domain to prevent neurotoxicity and fibril formation are dependent on a highly conserved Asp residue†

**DOI:** 10.1039/d2cb00187j

**Published:** 2022-09-15

**Authors:** Gefei Chen, Yuniesky Andrade-Talavera, Xueying Zhong, Sameer Hassan, Henrik Biverstål, Helen Poska, Axel Abelein, Axel Leppert, Nina Kronqvist, Anna Rising, Hans Hebert, Philip J. B. Koeck, André Fisahn, Jan Johansson

**Affiliations:** Department of Biosciences and Nutrition, Karolinska Institutet 141 52 Huddinge Sweden gefei.chen@ki.se; Neuronal Oscillations Laboratory, Center for Alzheimer Research, Departments of NVS and KBH, Karolinska Institutet 171 77 Stockholm Sweden; School of Engineering Sciences in Chemistry, Biotechnology and Health, Department of Biomedical Engineering and Health Systems, KTH Royal Institute of Technology 141 52 Huddinge Sweden; Department of Physical Organic Chemistry, Latvian Institute of Organic Synthesis Riga LV-1006 Latvia; School of Natural Sciences and Health, Tallinn University Tallinn Estonia; Department of Anatomy, Physiology and Biochemistry, Swedish University of Agricultural Sciences 750 07 Uppsala Sweden

## Abstract

Proteins can self-assemble into amyloid fibrils or amorphous aggregates and thereby cause disease. Molecular chaperones can prevent both these types of protein aggregation, but to what extent the respective mechanisms are overlapping is not fully understood. The BRICHOS domain constitutes a disease-associated chaperone family, with activities against amyloid neurotoxicity, fibril formation, and amorphous protein aggregation. Here, we show that the activities of BRICHOS against amyloid-induced neurotoxicity and fibril formation, respectively, are oppositely dependent on a conserved aspartate residue, while the ability to suppress amorphous protein aggregation is unchanged by Asp to Asn mutations. The Asp is evolutionarily highly conserved in >3000 analysed BRICHOS domains but is replaced by Asn in some BRICHOS families. The conserved Asp in its ionized state promotes structural flexibility and has a p*K*_a_ value between pH 6.0 and 7.0, suggesting that chaperone effects can be differently affected by physiological pH variations.

## Abstract 2

Activities of the BRICHOS domain against amyloid fibril formation and amyloid-induced neurotoxicity are affected by pH.

## Graphical abstract

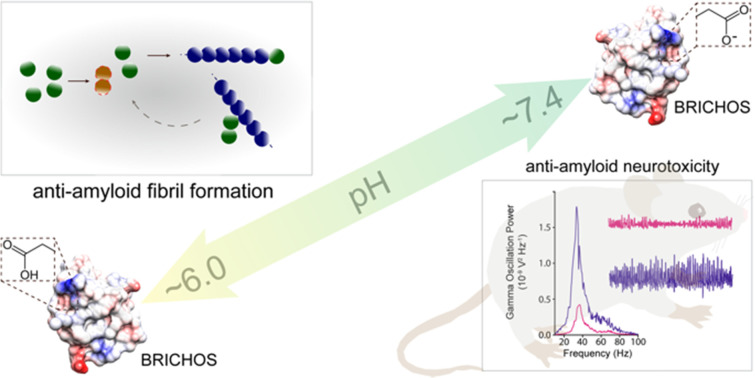

## Introduction

Proteins and peptides can self-assemble into highly ordered fibrillar structures as well as into smaller oligomers with less well-defined structures but apparently stronger ability to cause toxicity to cells. These phenomena are linked to amyloid diseases, which encompass about forty severe human diseases including interstitial lung disease (ILD), Alzheimer's disease (AD) and type 2 diabetes (T2D).^1,2^ Molecular chaperones prevent protein aggregation and cytotoxicity,^3^ and thus chaperone dysfunction as a result of *e.g.* ageing or mutations can lead to protein misfolding disorders.^4–6^ However, no clear correlation between the clinical phenotype and the severity of anti-aggregation defects of disease-causing molecular chaperone mutations has been shown for *e.g.* DNAJB6b and HSPB1.^7,8^ Amyloidogenic polypeptides can be expressed as proproteins which are subjected to proteolytic cleavage to release the amyloid forming fragments.^9–11^ Some amyloid-generating proproteins contain the domain BRICHOS, initially found in dementia-associated integral membrane protein 2B (ITM2B, also called Bri2), cancer-relevant Chondromodulin-1, and ILD-related prosurfactant protein C (proSP-C).^12,13^ The BRICHOS domain is suggested to promote the correct folding and prevent amyloid formation of the amyloid-prone region of its proprotein during biosynthesis,^14–16^ and is itself released by proteolysis.^17–19^

Recently, the BRICHOS domain has been shown to have chaperone activities against fibril formation and neurotoxicity of “alien” client peptides associated with human diseases but not part of BRICHOS containing proproteins,^19–21^ and has thus emerged as a model compound in studies of amyloid fibril formation.^22–25^ For instance, recombinant human (rh) BRICHOS domains recognise and bind to amyloid fibrils of amyloid β-peptide (Aβ, linked to AD) and islet amyloid polypeptide (IAPP, linked to T2D), and reduce their cellular toxicity,^25–27^ bind the smallest emerging toxic Aβ oligomers,^24^ bind to amyloid fibrils of Huntingtin (linked to Huntington's disease) and α-synuclein (linked to Parkinson disease),^28^ and can be used to efficiently reduce Aβ neurotoxicity in mouse hippocampal slice preparations and *in vivo* in animal models of AD.^27,29–33^ This broad anti-amyloid spectrum makes BRICHOS a potential candidate for amyloid disease prevention and even treatment.

Mutations in the BRICHOS domain or in the proproteins are associated with different protein misfolding and amyloid diseases,^14,34,35^ but the underlying pathogenic mechanisms are largely unknown. The rh BRICHOS domain from proSP-C is an efficient inhibitor of amyloid toxicity of Aβ42 in mouse hippocampal slice preparations and a *Drosophila* fruit fly model, reducing the generation of toxic Aβ42 oligomers, but it is not very competent to reduce the overall amyloid fibril formation rate.^20,23,31,32^ Rh Bri2 BRICHOS, on the other hand, assembles into differently sized species, of which monomers potently prevent Aβ42 neuronal toxicity, dimers strongly suppress Aβ fibril formation and large oligomers inhibit non-fibrillar protein aggregation.^20,30,32,36^ A Bri2 BRICHOS mutant (R221E), designed to stabilize the monomeric conformation prevents Aβ42 neurotoxicity rather than its overall fibrillization rate.^30^ A corresponding mutation in proSP-C BRICHOS (T187R) generates monomers that bind to the smallest emerging Aβ42 oligomers and is more efficient *in vitro* than the wildtype against amyloid fibril formation.^24^ Interestingly, another mutation at the same location in human proSP-C BRICHOS (T187N) leads to ILD with amyloid deposits.^37^ Thus, understanding the molecular mechanisms that regulate BRICHOS anti-amyloid chaperone activities are of interest from a basic science point of view, and for the development of treatments against amyloid diseases.

In the BRICHOS superfamily, the secondary structure elements are highly conserved but the amino acid sequence conservation is rather low, and only one aspartic acid residue (Asp, D) and two cysteine residues that form a disulphide bridge were previously found to be strictly conserved, from analyses of a rather small number of sequences.^12,30^ The physiological function of the Asp is unknown, yet two mutations of D105 in human proSP-C BRICHOS are linked to ILD.^14,18,38^ Here, we focused on this evolutionarily conserved Asp residue and investigated Asp to Asn mutations of two BRICHOS domains.

## Results

### The conserved aspartate is required for BRICHOS structural flexibility

**Fig. 1:**
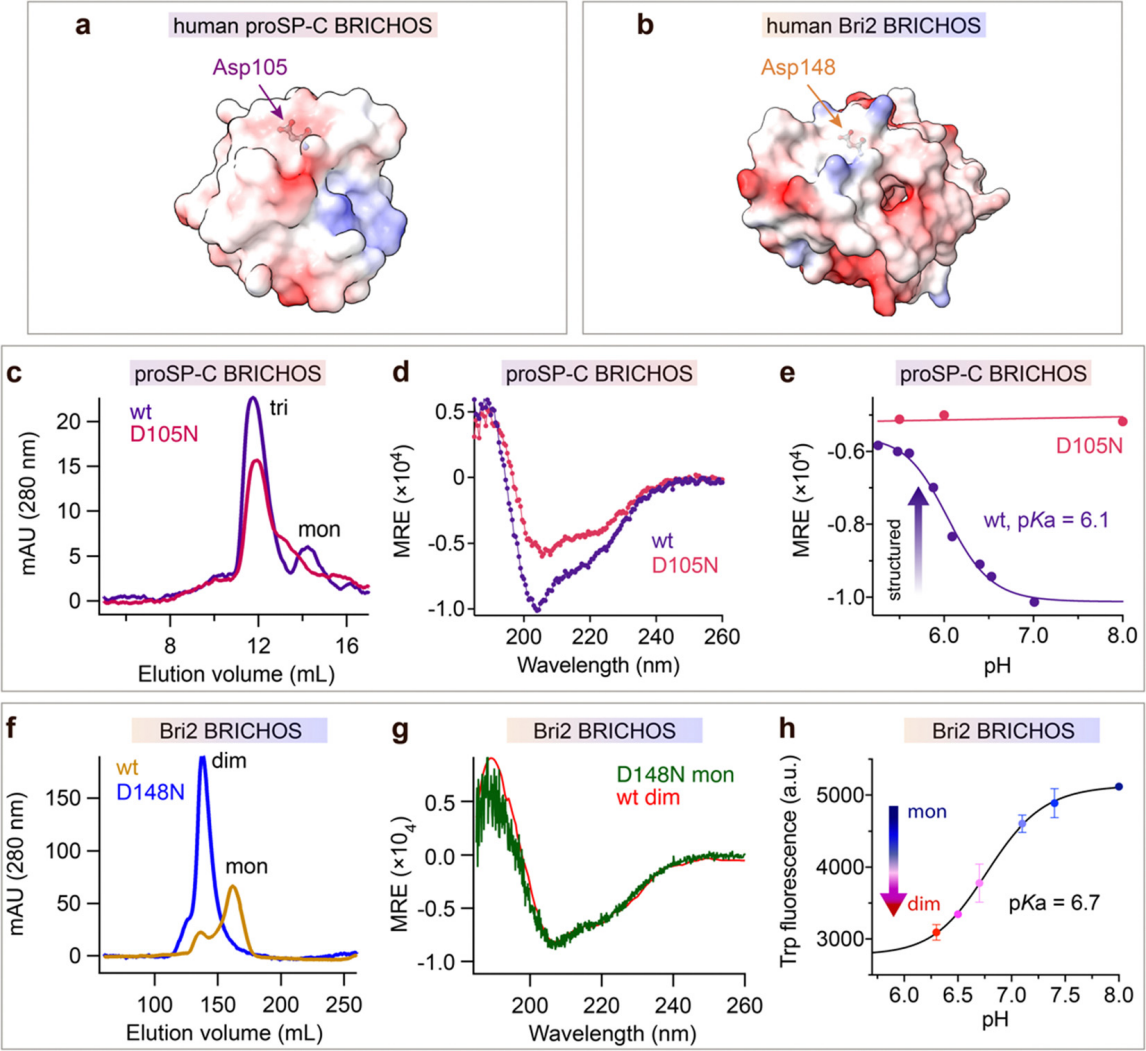
The conserved Asp in BRICHOS regulates the structural flexibility and titrates at physiological pH range. Electrostatic surface presentations (red-negative, blue-positive) of (a) human proSP-C BRICHOS monomer from crystal structure of trimer (pdb 2yad) and (b) monomeric human Bri2 BRICHOS structure predicated by AlphaFold.^76^ (c) SEC analysis of purified rh wildtype (wt) proSP-C BRICHOS (purple) and the D105N mutant (red). (d) CD spectra of rh wildtype (wt) proSP-C BRICHOS (purple) and the D105N mutant (red). (e) pH-dependent structural changes of rh proSP-C BRICHOS variants monitored by CD, the details are shown in ESI† Fig. S1a and b. (f) SEC of wildtype (wt) Bri2 BRICHOS (blue) and Bri2 BRICHOS D148N (yellow) monomer fraction prepared from corresponding fusion protein (NT*-Bri2 BRICHOS) monomers. dim, dimers; mon, monomers. (g) CD spectra of rh Bri2 BRICHOS D148N monomers and the comparison with rh wildtype (wt) Bri2 BRICHOS dimer. (h) pH-dependent transition of rh Bri2 BRICHOS monomers to dimers measured by Trp fluorescence, the details are shown in ESI† Fig. S5b and c. MRE is the mean molar residual ellipticity in deg cm^2^ dmol^−1^.

The observation that mutations of the conserved Asp in the BRICHOS domain can result in amyloid disease prompted us to mutate it to Asn. The BRICHOS domains from proSP-C (D105) and Bri2 (D148) were selected (Fig. 1a and b), since these two proteins are associated with different human diseases, *i.e.*, ILD and familial dementias, respectively, possess different chaperone activity spectra, and have only ∼17% amino acid sequence identities.^13,32,36^

Both wildtype and D105N rh proSP-C BRICHOS form trimers, but the mutant formed less monomers than the wildtype protein (Fig. 1c). Rh proSP-C BRICHOS D105N is overall more structured than the wildtype at neutral pH, while at acidic pH their secondary structures are virtually identical (Fig. 1d, ESI† Fig. S1a and b). The circular dichroism (CD) at 204 nm of rh wildtype proSP-C BRICHOS changed between pH 7.0 and 5.0, which gave an apparent p*K*_a_ of the transition of about 6.1 (Fig. 1e and ESI† Fig. S1a). Notably, the secondary structure of rh proSP-C BRICHOS D105N did not alter substantially between pH 5.5 and 8.0 (Fig. 1e and ESI† Fig. S1b), suggesting that protonation of D105 is a main determinant of the observed conformational changes, and it may be the sole titrating residue in this pH range. Nuclear magnetic resonance (NMR) spectroscopy of ^15^N/^13^C/^2^H labelled rh wildtype proSP-C BRICHOS confirmed the occurrence of structural changes between pH 7.2 and 5.5 (ESI† Fig. S1c).

Rh Bri2 BRICHOS D148N formed similar disulphide-dependent assembly states as the wildtype protein (ESI† Fig. S1d). Rh Bri2 BRICHOS D148N generated by cleavage from isolated rh NT*-Bri2 BRICHOS D148N monomers (NT* is a solubility tag^36,39^) migrated as a dimer on size-exclusion chromatography (SEC). In contrast, cleavage of differently sized NT*-Bri2 BRICHOS D148N species resulted in rh Bri2 BRICHOS D148N species with similar chromatographic profiles as the corresponding wildtype states (Fig. 1f and ESI† Fig. S1e). In line with this, rh Bri2 BRICHOS D148N oligomers and dimers shared identical secondary structure as the corresponding wildtype species, whereas the initially monomeric rh Bri2 BRICHOS D148N showed a somewhat different secondary structure compared to the wildtype monomers (ESI† Fig. S1f–h), and the CD spectrum of initially monomeric rh Bri2 BRICHOS D148N could be superimposed on that of the wildtype dimer (Fig. 1g). This suggests the rh Bri2 BRICHOS D148N monomer easily assembles into a dimer. Rh wildtype Bri2 BRICHOS at 1 μmol L^−1^ behaved as a monomer, and with increasing concentrations, dimers were progressively formed, but an exchange between dimers and monomers remained even at 200 μmol L^−1^ (ESI† Fig. S2a and S3). In contrast, already at low concentrations (0.5 μmol L^−1^) rh Bri2 BRICHOS D148N showed a distribution between dimers and monomers, approximately at a ratio of 1 : 1 (ESI† Fig. S2b and S4a). With progressively increased concentrations rh Bri2 BRICHOS D148N formed dimers and at 100 μmol L^−1^ only dimers were seen (ESI† Fig. S2b and S4). This indicates that the rh Bri2 BRICHOS D148N monomers are not stable and form non-covalent dimers. Further, the monomer–dimer transition was pH-dependent. At pH 7.0 and 8.0 rh Bri2 BRICHOS D148N and wildtype, respectively, showed similar elution volumes (ESI† Fig. S2c and d), but at pH 6.0 rh wildtype Bri2 BRICHOS eluted at a volume corresponding to a dimer, whereas the D148N mutant did not show any significant difference compared to the elution volume at pH 7.0 and 8.0 (ESI† Fig. S2c and d). This suggests that Asp148 of Bri2 BRICHOS can be protonated between pH 7.0 and 6.0 with subsequent shift of the monomer–dimer equilibrium towards dimers. To corroborate the supposition that Asp148 titrates between pH 6.0 and 7.0 we turned to a human Bri2 BRICHOS variant with Thr206 replaced by Trp (T206W). Rh Bri2 BRICHOS T206W displayed an identical oligomerization profile as the rh wildtype Bri2 BRICHOS (ESI† Fig. S5a), but its Trp fluorescence differed between the monomeric and dimeric states (ESI† Fig. S5b), thereby allowing monomer to dimer shifts to be followed by Trp fluorescence. Titration of rh Bri2 BRICHOS T206W showed a fluorescence evolution with a p*K*_a_ of 6.7 (Fig. 1h and ESI† Fig. S5c), supporting that the conserved Asp gets protonated between pH 6.0 and 7.0.

The above results suggest that the mutation D105N in rh proSP-C BRICHOS results in a more compact trimer conformation and that wt rh proSP-C BRICHOS assembles into a more compact conformation with an apparent p*K*_a_ of 6.1. Analogously, the D148N mutation in rh Bri2 BRICHOS promotes conversion from monomer to a more structured dimer, and mimics pH-induced dimerization of the wildtype protein with an apparent p*K*_a_ of 6.7. Taken together, these observations indicate that dynamically flexible structures of BRICHOS domain are maintained by the conserved Asp in its ironized state, and that more compact structures are formed upon Asp protonation.

### Aspartate to asparagine mutation abolishes the capacity to prevent amyloid neurotoxicity but enhances the ability to suppress fibril formation

To investigate whether the structural flexibility and the conserved Asp is important for function, we tested the efficacies of Asp to Asn mutated *vs.* wildtype BRICHOS in preventing Aβ42-induced reduction of *γ* oscillations in mouse hippocampal slices. *γ* oscillations correlate with learning, memory, cognition and other higher processes in the brain^40,41^ and progressive cognitive decline observed in AD goes hand-in-hand with a progressive decrease of *γ* oscillations.^42–44^ The recombinant BRICHOS domains from human proSP-C and Bri2 can efficiently prevent Aβ42-induced decline in hippocampal *γ* oscillations, observed for Aβ42 concentrations from 50 nmol L^−1^ to 1 μmol L^−1^.^23,30,32,36,45^ In addition, rh Bri2 BRICHOS rescues *γ* oscillations and neuronal network dynamics after Aβ42-induced impairment in hippocampal CA3 area.^46^

**Fig. 2:**
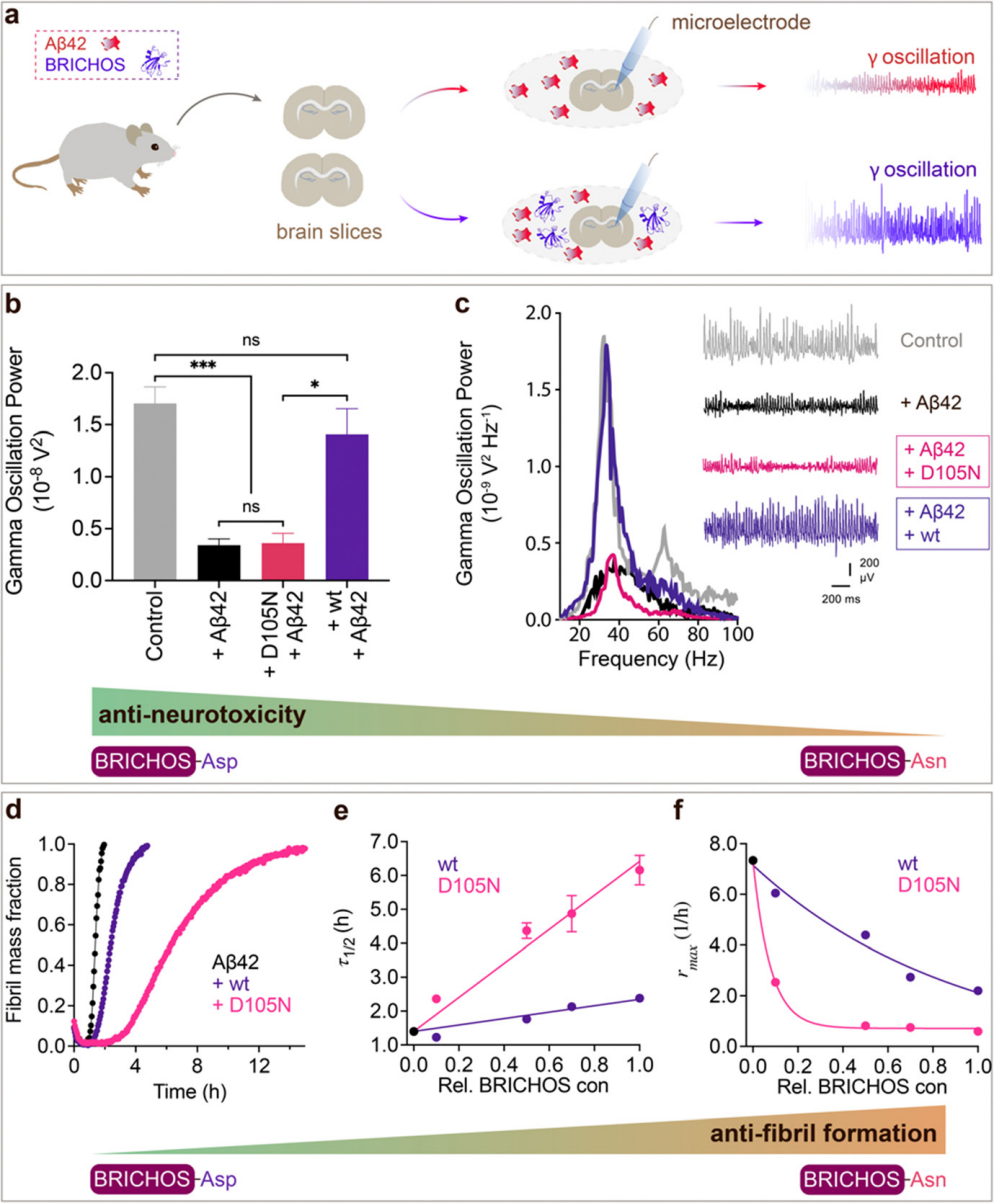
Effects of Asp to Asn mutation on rh proSP-C BRICHOS capacities against Aβ42 neurotoxicity and fibril formation, respectively. (a) Schematic diagram of electrophysiological recordings. The hippocampal slices from wildtype C57BL/6 mice were preincubated either with 50 nmol L^−1^ Aβ42 alone or co-incubated with 100 nmol L^−1^ rh BRICHOS, and γ oscillations were then recorded in the CA3 area. (b) Summary plot of normalized γ oscillation power under control conditions (gray, *n* = 20), after 15 min incubation with 50 nmol L^−1^ Aβ42 (black, *n* = 14), 50 nmol L^−1^ Aβ42 + 100 nmol L^−1^ D105N (red, *n* = 9) or wildtype (purple, *n* = 9) rh proSP-C BRICHOS. Example traces and example power spectra are shown in (c). The data are reported as means ± standard errors of the means. ns, no significant difference, *p < 0.05, **p < 0.01, ***p < 0.001. (d) Activity comparison of 100% rh wildtype proSP-C BRICHOS (purple) and rh proSP-C BRICHOS D105N (red) against 3 μmol L^−1^ Aβ42 (black). Individual fits with combined rate constants 
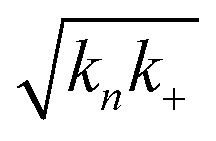
 and 
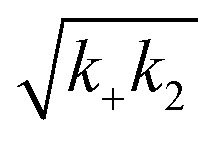
 as free fitting parameters of normalized and averaged aggregation traces (dots) are shown as solid lines. Values for *τ*_1/2_ (e) and *r*_max_ (f) extracted from sigmoidal fitting of Aβ42 aggregation traces in the presence of different concentrations of rh wildtype proSP-C BRICHOS (purple) or rh proSP-C BRICHOS D105N (red) as shown in (ESI† Fig. S6a–d). The triangles schematically indicate the relative activities of proSP-C BRICHOS with Asp and Asn at position 105.

We recorded *γ* oscillations in hippocampal slices from wildtype C57BL/6 mice preincubated for 15 min either with 50 nmol L^−1^ Aβ42 alone, or co-incubated with 100 nmol L^−1^ rh wildtype or D105N proSP-C BRICHOS (Fig. 2a). *γ* oscillations were elicited by application of 100 nmol L^−1^ kainic acid (KA) and allowed to stabilize for 30 min prior to any recordings. As previously observed,^23,32,45^ 100 nmol L^−1^ wildtype proSP-C BRICHOS prevented Aβ42-induced degradation of *γ* oscillations, which were remained at control levels (Fig. 2b and c, control: 1.7 ± 0.16 × 10^−8^ V^2^ Hz^−1^, *n* = 20; Aβ42: 0.34 ± 0.06 × 10^−8^ V^2^ Hz^−1^, *n* = 14, *p* < 0.0001 *vs.* control; + rh wildtype proSP-C BRICHOS: 1.41 ± 0.24 × 10^−8^ V^2^ Hz^−1^, *n* = 9). By contrast, surprisingly, rh proSP-C BRICHOS D105N showed a complete loss of the preventative efficacy at the same concentration (Fig. 2b and c, rh proSP-C BRICHOS D105N: 0.36 ± 0.09 × 10^−08^ V^2^ Hz^−1^, *n* = 9, *p* = 0.0202 *vs.* rh wildtype proSP-C BRICHOS, *p* = 0.0002 *vs.* control, *p* > 0.9999 *vs.* Aβ42). Apparently, mutating the conserved Asp to Asn in proSP-C BRICHOS dramatically impaired its capacity to prevent Aβ42-induced neurotoxicity.

To explore whether the abolished rh proSP-C BRICHOS capacity against Aβ42 neurotoxicity caused by Asp to Asn mutation correlates with the activity of suppressing macroscopic Aβ42 fibril formation, we used thioflavin T (ThT)^47^ fluorescence to monitor the kinetics of Aβ42 fibril formation in the absence and presence of different concentrations of rh proSP-C BRICHOS (Fig. 2d–f and ESI† Fig. S6a–d). Both the rh wildtype proSP-C BRICHOS and the D105N mutant showed dose-dependent progressive reduction of Aβ42 fibril formation at substoichiometric concentrations (Fig. 2d and ESI† Fig. S6a–d), and the final ThT fluorescence intensity did not change significantly in the presence of different concentrations of rh proSP-C BRICHOS variants (ESI† Fig. S6e). The Aβ42 fibrillization half time, *τ*_1/2_, increases linearly with increasing rh proSP-C BRICHOS concentration (Fig. 2e), while the maximum rate of Aβ42 aggregation, *r*_max_, shows a mono-exponential decline (Fig. 2f). Interestingly, rh proSP-C BRICHOS D105N showed improved inhibition of Aβ42 fibril formation compared to the wildtype, manifested for both *τ*_1/2_ and *r*_max_ (Fig. 2d–f), which is qualitatively opposite compared to the effects on the capacity to prevent Aβ42-induced neurotoxicity (Fig. 2a–c). These results show that the aspartate to asparagine mutation that induces a more compact conformation in rh proSP-C BRICHOS abolishes the capacity in preventing amyloid induced neurotoxicity, but significantly enhances the activity in prevention of amyloid fibril formation.

**Fig. 3:**
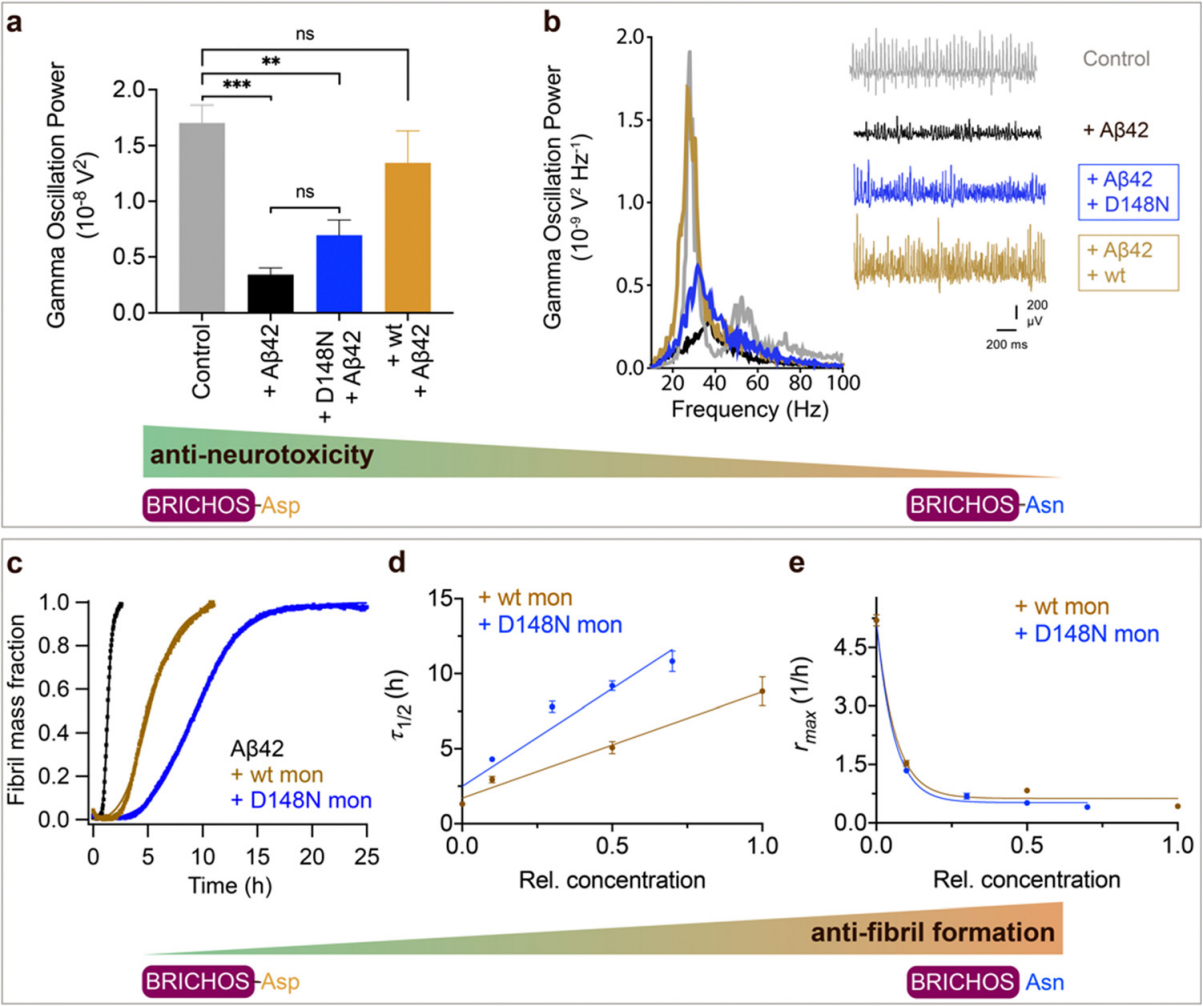
Effects of Asp to Asn mutation on rh Bri2 BRICHOS capacities against Aβ42 neurotoxicity and fibril formation, respectively. (a) Summary plot of γ oscillation power under control conditions (gray, *n* = 20), after 15 min incubation with 50 nmol L^−1^ Aβ42 (black, *n* = 14), 50 nmol L^−1^ Aβ42 + 100 nmol L^−1^ D148N monomeric (blue, *n* = 11) or wildtype monomeric (yellow, *n* = 8) rh Bri2 BRICHOS. Example traces and example power spectra are shown in (b). The data are reported as means ± standard errors of the means. ns, no significant difference, **p < 0.01, ***p < 0.001. (c) Comparison of 50% rh wildtype Bri2 BRICHOS (yellow) and rh Bri2 BRICHOS D148N (blue) activities against 3 μmol L^−1^ Aβ42 (black). The solid lines are from individual fits with combined rate constants 
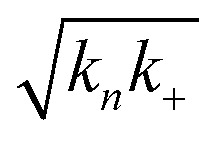
 and 
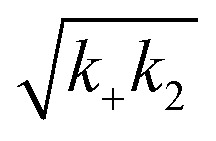
 as free fitting parameters of normalized and averaged aggregation traces (dots). Values for *τ*_1/2_ (d) and *r*_max_ (e) extracted from the sigmoidal fitting of Aβ42 aggregation traces in the presence of different concentrations of rh wildtype Bri2 BRICHOS and the D148N mutant as shown in ESI† Fig. S8. The triangles schematically indicate the relative activities of Bri2 BRICHOS with Asp and Asn at position 148.

We tested also the functional effects of rh wt and D148N Bri2 BRICHOS. We have previously observed that rh Bri2 BRICHOS monomer is most efficient at preventing Aβ42-induced degradation of *γ* oscillations^30,36^ and we therefore tested rh Bri2 BRICHOS D148N monomers. Rh Bri2 BRICHOS D148N monomers (50 nmol L^−1^) showed reduced potency to prevent Aβ42-induced neurotoxicity compared to wildtype monomers (50 nmol L^−1^), but did not completely lose its preventative efficacy (Fig. 3a and b, rh wildtype Bri2 BRICHOS monomers: 1.35 ± 0.29 × 10^−8^ V^2^ Hz^−1^, *n* = 8, *p* > 0.9999 *vs.* control; rh Bri2-BRICHOS D148N monomers: 0.7 ± 0.14 × 10^−8^ V^2^ Hz^−1^, *n* = 11, *p* = 0.3494 *vs.* rh wildtype Bri2 BRICHOS monomers, *p* = 0.003 *vs.* control, *p* = 0.6991 *vs.* Aβ42). In the ThT assay, rh Bri2 BRICHOS D148N monomers were significantly more efficient in inhibiting Aβ42 fibril formation compared to wildtype monomers (Fig. 3c–e).

### The canonical chaperone activity of BRICHOS is independent of the conserved Asp

**Fig. 4:**
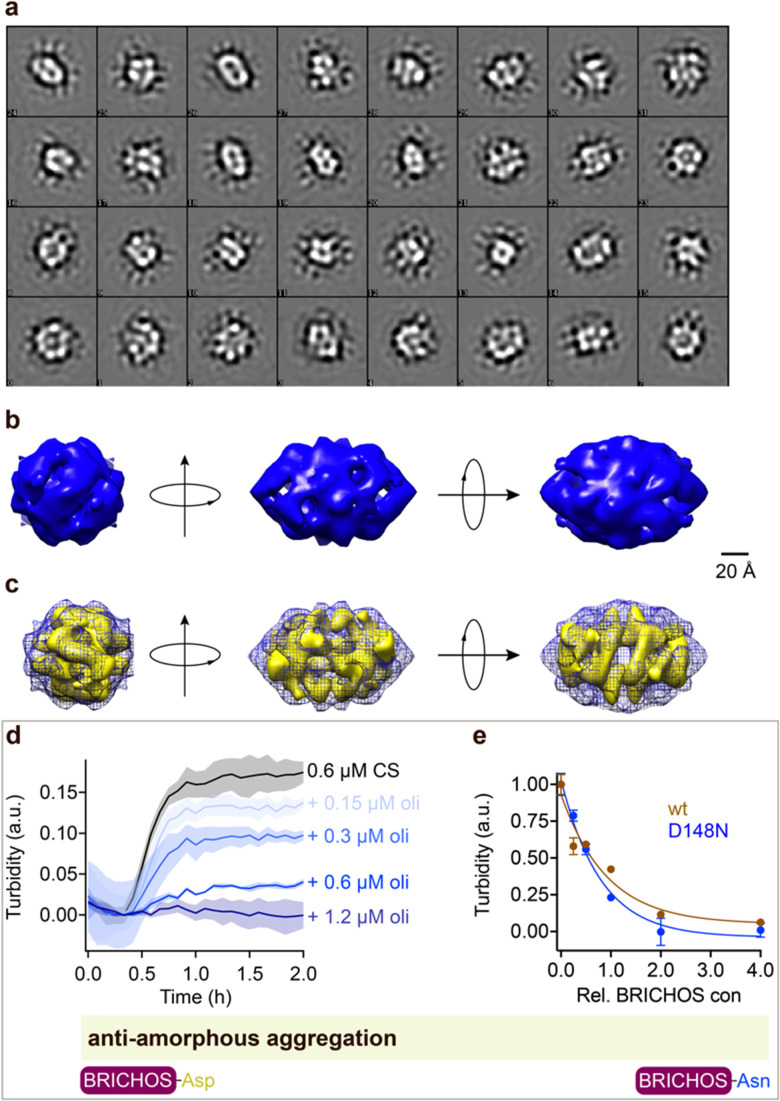
Rh Bri2 BRICHOS D148N oligomer 3D model construction and chaperone activity against amorphous protein aggregation. (a) 2D classes of rh Bri2 BRICHOS D148N oligomers. Most class averages show 2-fold symmetry. (b) The three viewing directions are along the three different 2-fold axes. The map of rh Bri2 BRICHOS D148N oligomer was based on 10 223 particles manually extracted from images recorded on a CCD detector with ×85 200 magnification and the voxel size of the map is 2.464 Å. (c) 3D density map of rh Bri2 BRICHOS D148N oligomer with D2 symmetry (blue grid) and the model of rh wildtype Bri2 BRICHOS oligomer (yellow, EMDB: 3918). (d) Kinetics of aggregation of 600 nmol L^−1^ citrate synthase (CS) at 45 °C alone (black), in the presence of 0.15, 0.3, 0.6 and 1.2 μmol L^−1^ rh Bri2 BRICHOS D148N oligomer. The different concentrations of the oligomers are shown with a blue gradient, which are labelled on the right of the traces. (e) Effects of rh Bri2 BRICHOS D148N and wildtype oligomers (data from ref. 36) on CS aggregation at different molar ratios (referred to monomeric subunits) of BRICHOS:CS. The data are presented as means ± standard deviations. The rectangle schematically indicates that the relative activities of Bri2 BRICHOS with Asp and Asn at position 105 are essentially identical.

Rh wildtype Bri2 BRICHOS oligomers are efficient molecular chaperones against amorphous, non-fibrillar, protein aggregation,^36,48^ while rh proSP-C BRICHOS lacks this function.^32^ Rh Bri2 BRICHOS D148N formed large oligomeric species with a similar secondary structure as wildtype oligomers (ESI† Fig. S1d–f). Transmission electron microscopy (TEM) of the rh Bri2 BRICHOS D148N oligomers and 2D class averages revealed homogenous large assemblies with two-fold symmetry (Fig. 4, ESI† Fig. S7a and b). An EM map was reconstructed with D2 symmetry from 10 223 manually extracted single particles (Fig. 4b). The rh Bri2 BRICHOS D148N and wildtype oligomer^36^ 3D models share overall similar shape and volume (Fig. 4c), suggesting that the overall structure of the large Bri2 BRICHOS oligomers is not noticeably changed when Asp148 is mutated to Asn. The rh Bri2 BRICHOS D148N oligomers are as efficient against amorphous protein aggregation as the wildtype oligomers, using thermo-induced citrate synthase aggregation as a model (Fig. 4d and e). This shows entirely different importance of the conserved Asp for activity against amyloid compared to amorphous protein aggregation, possibly *via* its effects on structural flexibility of BRICHOS domains.

### Mechanisms underlying different BRICHOS activities

**Fig. 5:**
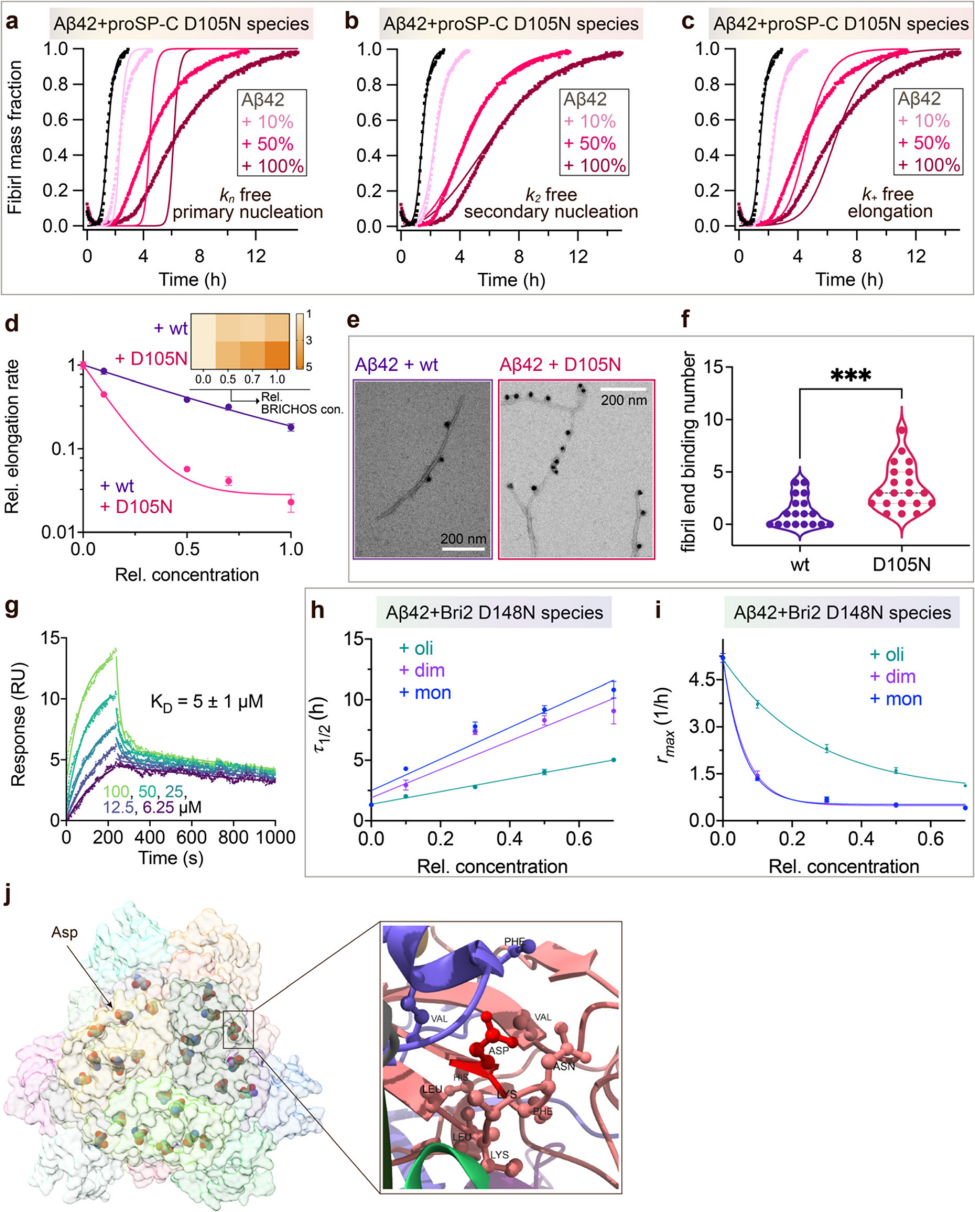
Mechanisms underlying BRICHOS uncoupled activities against neurotoxicity and fibril formation. (a–c) Aggregation kinetics of 3 μmol L^−1^ Aβ42 in the presence of rh proSP-C BRICHOS D105N at concentrations: 0 (black), 10 (light red), 50 (red), or 100% (dark red) molar percentage referred to monomeric subunits relative to Aβ42 monomer. The global fits (solid lines) of the aggregation traces (crosses) were constrained such that only one single rate constant is the free fitting parameter, indicated in each panel. *χ*^2^ values describing the quality of the fits: 18 for *k*_*n*_ free, 0.8 for *k*_2_ free and 3.2 for *k*_+_ free. (d) Elongation rates (*k*_+_) determined from highly seeded aggregation kinetics in ESI† Fig. S6f and g. The inset shows the amount of toxic Aβ42 oligomers calculated with the relative elongation rates (*k*_+_) and secondary nucleation rates (*k*_2_) for either rh wildtype proSP-C BRICHOS or the D105N mutant. (e) Immuno-EM of BRICHOS bound to Aβ42 fibrils. Aβ42 was incubated with and without 100% molar ratio of rh wildtype proSP-C BRICHOS and the D105N mutant, respectively, overnight at 37 °C. The samples were treated with a polyclonal antibody against human proSP-C and a gold-labelled secondary antibody and characterized by TEM. The scale bars are 200 nm. (f) Quantification of the fibril-end binding BRICHOS molecules per micrograph analysed. ***p < 0.001. (g) SPR sensorgrams of different concentrations (*i.e.*, 6.25, 12.5, 25, 50, and 100 μmol L^−1^) of rh proSP-C BRICHOS D105N interacting with immobilized Aβ42 monomers. The data were globally fitted for the association and disassociation phases, respectively, and the apparent *K*_D_ was calculated. (h–i) Values for *τ*_1/2_ (h) and *r*_max_ (i) extracted from the sigmoidal fitting of Aβ42 aggregation traces in the presence of different concentrations of rh Bri2 BRICHOS D148N species as shown in in ESI† Fig. S8a–i. (j) Cryo-EM model of rh Bri2 BRICHOS oligomers (EMDB: 7Q8X). Locations of the conserved Asp is show in ball and stick and exemplified by the arrow. The surrounding amino acid residues (distance less than 5 Å to the Asp) are shown in ball and stick (right panel).

To investigate what mechanisms that might underlie the opposite effects of mutating the conserved Asp on overall amyloid fibril formation and neurotoxicity, we investigated microscopic events of Aβ42 fibrillization in the absence and presence of wildtype or mutant BRICHOS. Aβ42 fibrillization kinetics can be described by different microscopic rate constants, *i.e.*, *k*_*n*_ for primary, *k*_2_ for surface-catalyzed secondary nucleation as well as *k*_+_ for elongation,^49^ and perturbations of these individual microscopic rates determine the generation of nucleation units, which might be linked to the formation of neurotoxic Aβ42 oligomeric species.^23,50^ We performed global fits of the kinetics traces at constant Aβ42 and different BRICHOS concentrations for both rh wildtype proSP-C and the D105N mutant (Fig. 5a–c and ESI† Fig. S6b–d). As previously described,^23^ the rh wildtype proSP-C BRICHOS mainly interfered with the secondary nucleation, indicated by the perfect fits when *k*_2_ was the sole global fitting rate constant (ESI† Fig. S6b–d). Also, the secondary nucleation rate *k*_2_ as the sole fitting parameter gave the best fits for the Aβ42 fibrillization kinetics with rh proSP-C BRICHOS D105N (Fig. 5a–c), but with worse quality compared to the wildtype, especially for the start of the aggregation traces. This suggests that a complex microscopic mechanism is present for rh proSP-C BRICHOS D105N, probably including fibril-end elongation (*k*_+_) and/or primary nucleation (*k*_*n*_) in addition to secondary nucleation (*k*_2_). However, the effects on secondary nucleation (*k*_2_) of rh wildtype proSP-C and the D105N mutant did not show striking differences (ESI† Fig. S6f). The complex microscopic mechanism was further analyzed by seeding experiments^49^ and surface plasmon resonance (SPR). With seeding, the fibrillization traces typically follow a concave aggregation behavior (ESI† Fig. S6g and h), where the initial slope is directly proportional to the elongation rate *k*_+_. The seeding experiments revealed that rh proSP-C BRICHOS D105N decreases the elongation in a dose-dependent manner, and already at low concentrations fibril-end elongation is noticeably retarded (Fig. 5d and ESI† Fig. S6h). The rh wildtype proSP-C BRICHOS, in contrast, showed only slight effects on fibril-end elongation (Fig. 5d and ESI† Fig. S6g). This difference is further supported by immuno-transmission electron microscopy (immuno-EM) images where both rh proSP-C BRICHOS D105N and the wildtype attach along the Aβ42 fibril surface, while the fibril ends are apparently mainly blocked by the D105N mutant (Fig. 5e and f). Additionally, SPR analyses indicated that rh wildtype proSP-C BRICHOS showed only weak binding to immobilized Aβ42 monomers, in line with previous reports.^23,24^ The D105N mutation significantly enhanced the BRICHOS-Aβ42 monomer interactions (ESI† Fig. S6i) with an apparent *K*_D_ value around 5 μmol L^−1^ (Fig. 5g) from global kinetics fits, and under steady-state conditions a *K*_D_ value of around 25 μmol L^−1^ was obtained (ESI† Fig. S6j). Interfering with primary nucleation (*k*_*n*_) delays Aβ42 fibril formation without changing the total number of oligomers generated, while suppressing the secondary nucleation (*k*_2_) efficiently prevents the generation of oligomers, and blocking elongation (*k*_+_) significantly increases the number of oligomers formed.^23^ Of relevance for the results on Aβ42 neurotoxicity of wildtype *vs.* D105N proSP-C BRICHOS (Fig. 2 and 3), we found that in the presence of equimolar ratio of rh proSP-C BRICHOS D105N, the Aβ42 fibrillization reaction generated approximately 5.2 times more oligomers than from Aβ42 alone, by significantly suppressing the fibril end elongation process, while the wildtype only showed slight effects (Fig. 5d inset). These observations show that both rh proSP-C BRICHOS D105N and the wildtype protein reduce Aβ42 fibrillization by blocking the surface-catalyzed secondary nucleation, while fibril-end elongation and primary nucleation are substantially affected only by the D105N mutant. These differences offer a possible molecular explanation to the observed loss of inhibitory effects of the rh proSP-C BRICHOS mutant on Aβ42 neurotoxicity.

For rh Bri2 BRICHOS, dimers were found previously to be most efficient in preventing Aβ42 overall fibril formation compared to the monomers and oligomers, while the monomers are most potent in preventing Aβ42 induced disruption of neuronal network activity.^30,36^ The rh Bri2 BRICHOS D148N oligomers, dimers and monomers presented dose-dependent inhibition on Aβ42 fibril formation with typical sigmoidal behavior (Fig. 3c, 5h and i, ESI† Fig. S8), and did not obviously change the final ThT fluorescence intensities (ESI† Fig. S8j). Also, the microscopic mechanisms of rh Bri2 BRICHOS D148N species against Aβ42 fibrillization are similar to the wildtype species,^36,51^ both the secondary nucleation and elongation of Aβ42 are affected (ESI† Fig. S8a–i). However, compared to wildtype monomers, the D148N monomers were significantly more efficient in inhibiting Aβ42 fibril formation (Fig. 3c–e), and D148N monomers and dimers showed very similar inhibitory effects on Aβ42 fibrillization (Fig. 5h and i). This can likely be explained by that rh Bri2 BRICHOS D148N monomer–dimer equilibrium is shifted towards the dimer for the mutant, but not the wt protein (ESI† Fig. S2–S4). These results suggest that the Asp to Asn mutant does not qualitatively modify the underlying mechanism of rh Bri2 BRICHOS, but enhances the activity of inhibiting Aβ42 fibrillization, and diminishes the anti-Aβ42 neurotoxicity ability by promoting formation of the dimer over the monomer.

All the so far studied BRICHOS domains are able to suppress amyloid fibril formation, but the activity against amorphous protein aggregation is only found for oligomers of rh Bri2 and Bri3 BRICHOS.^32,36,52^ Recently, we constructed a 3D structure model of rh Bri2 BRICHOS 24-mers from single-particle cryogenic EM data (EMDB: 7Q8X), which shows that the conserved Asp148 is buried and not involved in intersubunit interactions (Fig. 5j). This is in line with the result that rh Bri2 BRICHOS D148N forms large oligomers as the wildtype protein (ESI† Fig. S1d and e). The rh Bri2 BRICHOS activity to suppress amorphous protein aggregation was found to be dependent on three solvent exposed loop segments that are distant from Asp148 (ESI† Fig. S7c) (Chen *et al.*, submitted for publication). These observations provide a likely explanation for the different effects the rh Bri2 BRICHOS D148N mutation on anti-amyloid activity *versus* ability to suppress amorphous protein aggregation.

### Evolutionary conservation of Asp in BRICHOS domains

**Fig. 6:**
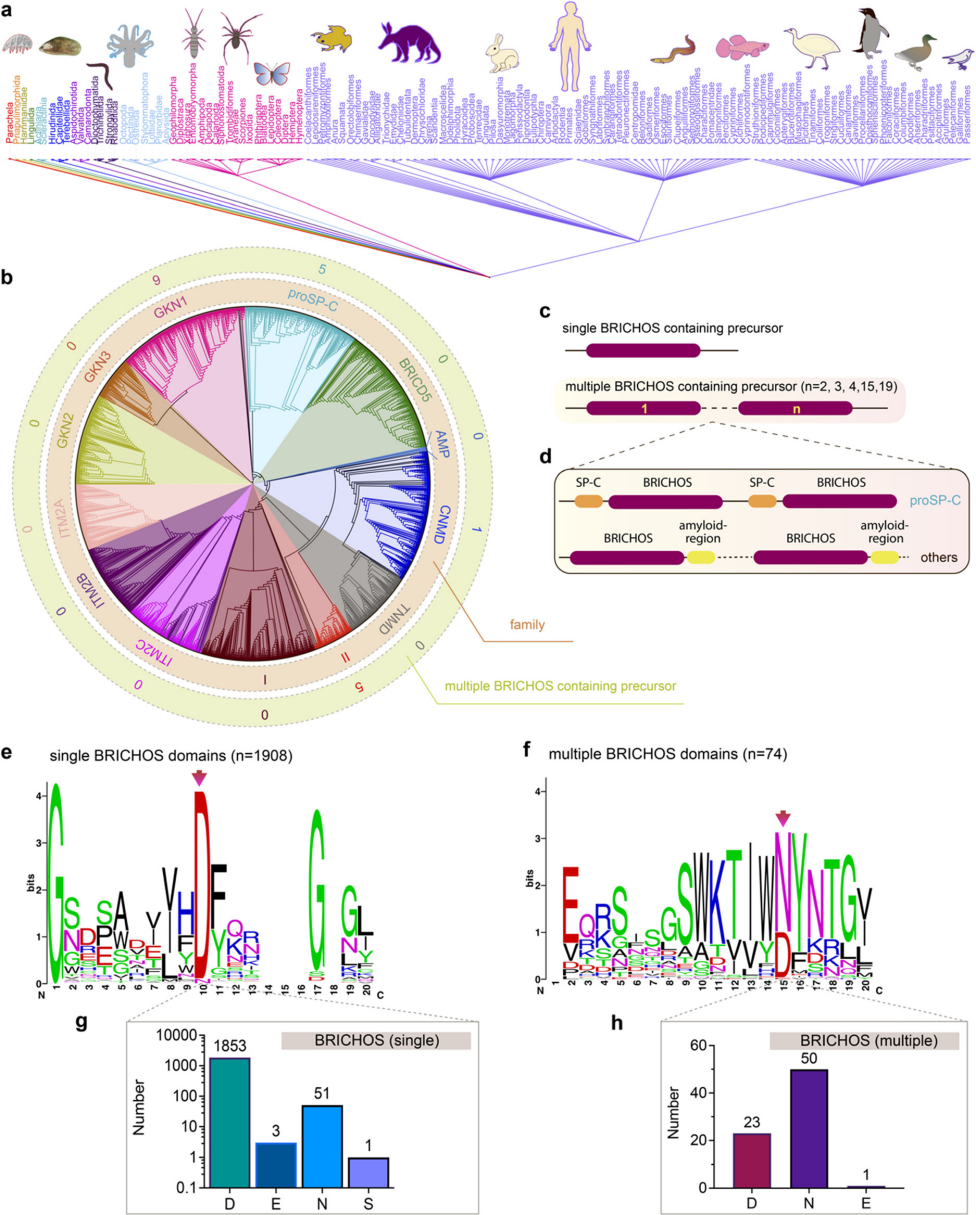
Evolutionary analyses of the BRICHOS domain. (a) The common taxonomy tree for all species containing BRICHOS domain precursors. The 3355 BRICHOS precursors are distributed in a broad range of species, including worms, insects to human. (b) The selected 2019 BRICHOS domains are grouped into thirteen families (inner ring), with bootstraps shown in ESI† Fig. S9. The outer ring shows the number of cases of occurrence of multiple BRICHOS domains in the respective families. (c and d) Architecture of proproteins containing single and up to nineteen multiple BRICHOS domains. The detailed architectures for each case are shown in ESI† Fig. S10. (d) Proproteins that contain multiple BRICHOS domains along with representatives of their corresponding amyloid-prone regions. The true domain size is not proportional to the schematic bar. (e) WebLogo representation of amino acid sequence alignments of the 1908 single BRICHOS domains, surrounding the conserved Asp (red arrow). The height of the amino acid stack (bits) represents the sequence conservation, while the height of symbols within each stack indicates the relative frequency of each residue. The exact residue distribution at the position of the conserved Asp is shown at the bottom (g). (f) WebLogo representation and exact residue distribution at the position of the conserved Asp as in panel (c) of sequence alignments of the 74 multiple BRICHOS domains. The exact residue distribution is shown at the bottom (h).

The unexpected diverse effects of mutating the conserved Asp to Asn in proSP-C and Bri2 BRICHOS inspired us to analyse all so far deposited BRICHOS containing proproteins. Amino acid sequences of 3355 BRICHOS domain precursors were extracted from the SMART database, covering 537 different metazoan species spanning worms to humans (Fig. 6a). After sorting out repetitive and incomplete sequences, 2019 BRICHOS sequences from 1968 precursor proteins were further analysed. These BRICHOS domains were separated into thirteen groups, which include 11 previously known families (integral membrane protein 2A (ITM2A), ITM2B, ITM2C (also called Bri1, Bri2 and Bri3, respectively), group I, gastrokine-1 (GKN1), GKN2, GKN3, tenomodulin (TNMD), chondromodulin (CNMD, previously called LECTI), proSP-C, BRICHOS containing domain 5 (BRICD5)) and two novel families (group II and antimicrobial peptide (AMP)) (Fig. 6b centre and inner ring, ESI† Fig. S9). The BRICHOS domains in the AMP group were recently found to help antimicrobial peptides to fold correctly in marine animals,^53–55^ while the functions of BRICHOS domains in group II are unknown.

Interestingly, 1% of all analysed BRICHOS containing proproteins contain multiple BRICHOS domains along with their corresponding amyloid-prone regions, and these were found in GKN1, proSP-C, CNMD and group II families (Fig. 6b outer ring, Fig. 6c and d and ESI† Fig. S10). Up to nineteen BRICHOS domains were found in one precursor (ESI† Fig. S10). The multiple BRICHOS domains within the same precursor showed high pairwise identities ranging from 48% to 97% (ESI† Table S1). Alignment of all compiled BRICHOS amino acid sequences showed that the Asp residue, which previously was found to be strictly conserved, did not show 100% conservation in neither the single nor the multiple BRICHOS domains (Fig. 6e and f, ESI† Fig. S11 and S12). In the single BRICHOS domains, 97% contain the conserved Asp, while 2.7% have Asn instead, and the remaining 0.3% are distributed between Glu (E) and Ser (S) (Fig. 6g). Among the multiple BRICHOS domains, the percentage with Asp was decreased to 31%, while 68% contain Asn, and one example with Glu was found (Fig. 6h). Among the single BRICHOS domains, GKN1, GKN3, ITM2A, and proSP-C families contain Asn, while, surprisingly, all the non-Asp BRICHOS domains from the precursors with multiple BRICHOS domains were found in the GKN1 family.

### Discussion

In this work, we find that the capacity of the BRICHOS domain to inhibit amyloid-associated neurotoxicity and fibril formation, respectively, is oppositely affected by mutations of a phylogenetically conserved Asp, whereas the capacity to suppress non-fibrillar, amorphous protein aggregation is not affected by Asp to Asn replacement. Moreover, conformational changes occur as a result of Asp to Asn mutations in both proSP-C and Bri2 BRICHOS, and those changes can partly be mimicked by lowered pH and the conserved Asp titrates with an apparent p*K*_a_ between 6 and 7 in both BRICHOS domains studied.

The Asp residue studied herein is the only non-Cys residue conserved in most known BRICHOS domains. Two mutations of this residue in human proSP-C BRICHOS (D105) are linked to ILD.^14,18,38^ Based on molecular dynamic simulations, monomeric wildtype proSP-C BRICHOS and a D105N mutant behaved differently: only minor conformational changes were seen in the mutant, but several large-scale changes occurred in the wildtype protein at moderately elevated temperatures, which resulted in a more loosely folded structure.^14^ D105N mutation in rh proSP-C BRICHOS results in a more ordered conformation, as judged by CD spectroscopy, and apparently more efficient trimer formation, while D148N mutation of rh Bri2 BRICHOS results in transition of monomers to more compact dimers (Fig. 1c, d, f and g). Similar effects as observed for the mutants were seen for the wildtype proteins when pH was lowered to 6–7 (Fig. 1e and f). This suggests that a negatively charged Asp sidechain is necessary for maintaining a “loose” flexible state of the BRICHOS subunit and that protonation, or replacement with an Asn, results in a more “compact” state. In the loose conformation, BRICHOS is efficient in alleviating Aβ42 amyloid neurotoxicity, while the compact conformation is more potent against overall amyloid fibril formation but inefficient against amyloid induced neurotoxicity (Fig. 2 and 3). The distant evolutionary relationship between Bri2 and proSP-C BRICHOS domains, with ∼17% sequence identities, makes it possible that the common effects now observed between them also apply to other BRICHOS domains that exhibit similar evolutionary distances. Therefore, an elevated p*K*_a_ value of the only highly conserved non-Cys residue, as now found for proSP-C and Bri2 BRICHOS, is probably relevant for a common function of all BRICHOS domains. Our results indicate that such a hypothetical common function is likely to be related to prevention of amyloid toxicity. Interestingly, in some cases, in particular when multiple BRICHOS domains are present, the conserved Asp is replaced with Asn (Fig. 6).

Molecular chaperones or chaperone-like domains have not been studied extensively as regards sensitivity to pH. One exception is clusterin, the activity of which is enhanced at mildly acidic pH, which appears to result from an increase in regions with solvent-exposed hydrophobicity, but independent of any major changes in secondary or tertiary structure.^56^ It has been shown that the small heat shock protein αB-crystallin uses different interfaces to bind to amyloid and amorphous substrates, respectively, but it is not known how different conformational states of αB-crystallin are regulated to affect activities against different types of substrates.^57^ The chaperoning capacities of BRICHOS domain against amyloid neurotoxicity and fibril formation can apparently be modulated by a conserved Asp in response to pH changes, suggesting the possibility that the microenvironment may affect BRICHOS function. For example, pH in the secretory pathway span from 7.2 to 5.7.^58^ The results here are based on *in vitro* and *ex vivo* experiments, and further work that addresses the different activities of BRICHOS are motivated.

## Materials and methods

### Phylogenetic analysis of BRICHOS domain

From the SMART database,^59^ 3355 BRICHOS sequences were downloaded while the sole bacterial BRICHOS precursor (*Paeniclostridium sordellii* ATCC 9714) was not included. Incomplete sequences were filtered out, resulting in total of 3190 sequences. Identical sequences were filtered by means of CD-HIT,^60^ with a threshold of 100% of sequence identity, which gave 3093 amino acid sequences. The hidden markov model profile (HMM) of BRICHOS (PF04089) was extracted from PFAM database.^61^ The CD-HIT filtered BRICHOS protein sequences were then scanned against the HMM profile using HMMER software v3.3.2^62^ with an *E*-value cut-off less than 1.0 × 10^−5^. An in-house python script was written to filter out the significant BRICHOS proteins from the HMM result file and to extract the BRICHOS domain sequences (each BRICHOS amino acid sequence was extended six residues upstream from the BRICHOS domain starting position defined by PFAM). Further, BRICHOS domain sequences less than 68 aa were removed as one rodent BRICHOS domain is just 69 residues and still functional against amyloid fibril formation,^63^ which eventually generated 2019 BRICHOS sequences. The multiple sequence alignment of the 2019 BRICHOS domain sequences were created using MAFFT alignment server with the default settings,^64^ and the sequence logo was generated using Weblogo webserver.^65^ RAxML HPC (v8.2.10)^66^ was employed for constructing the phylogenetic tree using the PROTGAMMAAUTO model with 100 times bootstrap iterations. The tree shown in this study was visualized using the Interactive Tree of Life (iTOL) server^67^ and Geneious software. The taxonomy tree common for species that contain BRICHOS precursors were generated by NCBI Taxonomy (https://www.ncbi.nlm.nih.gov/Taxonomy/CommonTree/wwwcmt.cgi) and visualized by Geneious software.

### Rh Bri2 BRICHOS and rh proSP-C BRICHOS wildtype and mutant preparation

For generating rh Bri2 BRICHOS D148N, the amplification primers 5′- ATAGTGATCCTGCCAACATTGATAACTTTAACAAGAAACTTACA-3′ and 5′- TGTAAGTTTCTTGTTAAAGTTATGAACAATGTTGGCAGGATCACTAT-3′ were synthesized. With the wildtype NT*-Bri2 BRICHOS (corresponding to the solubility tag NT* followed by Bri2 residues 113–231^36,39^) plasmid as PCR template Bri2 BRICHOS D148N was obtained with QuikChange II XL Site-Directed Mutagenesis Kit (Agilent, US), and the DNA sequence was confirmed (GATC Bioteq, Germany). Similarly, the amino acid Thr at position 206 was mutated to Trp using KAPA HiFi HotStart ReadyMix PCR Kit (Kapa Biosystems, USA) together with the designed complementary primers (5′-CCTATCTGATTCATGAGCACATGGTTATTTGGGATCGCATTGAAAAC-3′ and 5′-GTTTTCAATGCGATCCCAAATAACCATGTGCTCATGAATCAGATAGG-3′) and verified by sequencing (Eurofins Genomics). As described,^30,36^ the Bri2 BRICHOS variants were expressed in SHuffle T7 *E. coli* cells. Briefly, the cells were incubated at 30 °C in LB medium with 15 μg mL^−1^ kanamycin until an OD_600nm_ around 0.9. For overnight expression, the temperature was lowered to 20 °C, and 0.5 mmol L^−1^ (final concentration) isopropyl β-d-1-thiogalactopyranoside (IPTG) was added. The induced cells were harvested by centrifugation (4 °C, 7000 × *g*) and the cell pellets were resuspended in 20 mmol L^−1^ Tris pH 8.0. After 5 min sonication (2 s on, 2 s off, 65% power) on ice, the lysate was centrifuged (4 °C, 24 000 × *g*) for 30 min and the protein of interest was isolated with a Ni-NTA column. To remove the His_6_-NT* part, the target proteins were cleaved with thrombin (1 : 1000, w/w) at 4 °C overnight and loaded over a second Ni-NTA column. Different species of rh Bri2 BRICHOS variants were isolated and analysed by size-exclusion chromatography (SEC) with Superdex 200 PG, 200 GL or 75 PG columns (GE Healthcare, UK) using an ÄKTA system (GE Healthcare, UK) with buffer of 20 mmol L^−1^ NaPi (Sodium Phosphate) with 0.2 mmol L^−1^ EDTA at different pHs. For generating rh proSP-C BRICHOS D105N, the PCR primers 5′-CACTGGCCTCGTGGTGTATAACTACCAGCAGCTGCTGATCGC-3′ and 5′- GCGATCAGCAGCTGCTGGTAGTTATACACCACGAGGCCAGTG-3′ were synthesized. With the wildtype proSP-C BRICHOS (corresponding to the solubility tag thioredoxin followed by proSP-C residues 86–197^14^) plasmid as PCR template, proSP-C BRICHOS D105N was obtained with QuikChange II XL Site-Directed Mutagenesis Kit (Agilent, US), and the DNA sequence was confirmed (GATC Bioteq, Germany). The expression and purification were performed as described.^14,68^ Briefly, both wildtype proSP-C BRICHOS and the D105N mutant were expressed in Origami 2 (DE3) pLysS *E. coli* cells. The cells were grown at 37 °C in LB medium containing 100 μg mL^−1^ ampicillin until an OD_600nm_ around 0.9. The temperature was lowered to 25 °C and 0.5 mmol L^−1^ (final concentration) IPTG was added for overnight expression. The cells were harvested by centrifugation at 7000 × *g* for 20 min, and the cell pellets were resuspended in 20 mmol L^−1^ Tris pH 8.0. The protein was purified using Ni-NTA column and ion exchange chromatography (QFF, GE Healthcare). Thrombin (1 : 1000, w/w) was used to remove the thioredoxin tag. The purified rh proSP-C BRICHOS variants were analysed by Superdex 200 GL columns (GE Healthcare, UK) using an ÄKTA system (GE Healthcare, UK). The BRICHOS mutants in this study were expressed and purified in parallel with their wildtype counterparts.

### NMR spectroscopy

For the NMR experiments, gene fragment encoding human proSP-C BRICHOS was transformed into SHuffle T7 *E. coli* cells and was grown in LB with gradually increasing D_2_O content (25%, 50%, 75% and 100%). At 100% D_2_O, 1 mL LB was used to inoculate 100 mL M9 in 100% D_2_O enriched with ^15^N H_4_Cl and ^13^C glucose, and was grown over night at 31 °C. After overnight incubation, the 100 mL was added to 900 mL M9 in D_2_O enriched with ^15^N and ^13^C and grown at 30 °C until OD_600nm_ was around 0.8. The temperature was lowered to 20 °C and 0.5 mmol L^−1^ (final concentration) IPTG was added for overnight expression. The purification was performed as described above.

2D ^1^H–^15^N TROSY-HSQC experiments were obtained at 37 °C on Bruker 800 MHz spectrometer equipped with a TCI cryogenic probe. Spectra were processed with the software NMRPipe and visualized using Sparky NMR. The concentrations of ^2^H, ^15^N,^13^C-labeled proSP-C was 288 μmol L^−1^ in 20 mmol L^−1^ ammonium acetate pH 7.2 and 229 μmol L^−1^ in 20 mmol L^−1^ ammonium acetate pH 5.5, both in 90% H_2_O/10% D_2_O.

### Circular dichroism and fluorescence spectroscopy and aggregation analyses

Circular dichroism (CD) spectra were recorded in 1 mm path length quartz cuvettes at 25 °C from 260 to 185 nm in a J-1500 Circular Dichroism Spectrophotometer (JASCO, Japan) with a protein concentration of 4.2–10 μmol L^−1^. The bandwidth was set to 1 nm, data pitch 0.5 nm, and scanning speed 50 nm min^−1^. The spectra shown are averages of five consecutive scans.

Citrate synthase from porcine heart (Sigma-Aldrich, Germany) was diluted in 40 mmol L^−1^ HEPES/KOH pH 7.5 to 600 nmol L^−1^ (calculated from a molecular weight of 85 kDa corresponding to a dimer^69^) and then equilibrated at 45 °C with and without different concentrations of rh Bri2 BRICHOS D148N oligomer or proSP-C BRICHOS variants. The aggregation kinetics were measured by reading the apparent increase in absorbance at 360 nm under quiescent conditions using a microplate reader (FLUOStar Galaxy from BMG Labtech, Offenberg, Germany).

Different rh BRICHOS (1 μmol L^−1^, calculated for the monomeric subunit) in 20 mmol L^−1^ NaPi pH 8.0 or pH 6.0 were incubated at 25 °C with 2 μmol L^−1^ bis-ANS (4,4′-bis(phenylamino)-[1,1′-binaphthalene]-5,5′-disulfonic acid dipotassium salt) for 10 min, and the fluorescence emission spectra were recorded from 420 to 600 nm after excitation at 395 nm with the Infinite M1000 plate reader (Tecan, Austria). Rh Bri2 BRICHOS T206W monomers were diluted to 2 μmol L^−1^ by using 20 mmol L^−1^ NaPi containing 0.2 mmol L^−1^ EDTA with different pH in the final samples in the range of 6.3–8.0. For tryptophan fluorescence measurements, samples were prepared in duplicates with a volume of 150 μL. Samples were excited at 280 nm (5 μm bandwidth) and fluorescence emission from 300–400 nm (10 μm bandwidth, 1 nm step interval) was recorded on black polystyrene flat-bottom 96-well plates (Costar) using a spectrofluorometer (Tecan Saphire 2). For the final fluorescence intensities, the results were corrected by subtracting the background fluorescence of the buffer.

### Transmission electron microscopy imaging of rh Bri2 BRICHOS D148N oligomers and single particle processing

Rh Bri2 BRICHOS D148N oligomers after SEC isolation were immediately stored on ice followed by grid preparation. Aliquots (4 μL) were adsorbed onto glow-discharged continuous carbon-coated copper grids (400 mesh, Analytical Standards) for one min. The grids were subsequently blotted with filter paper, washed with two drops of Milli-Q water, and negatively stained with one drop of 2% (w/v) uranyl acetate for 45 s before final blotting and air-drying. The sample was imaged using a Jeol JEM2100F field emission gun transmission electron microscope (Jeol, Japan) operating at 200 kV. Single micrographs for evaluating the quality of the sample were recorded on a Tietz 4k × 4k CCD camera, TVIPS (Tietz Video and Image Processing Systems, GmbH, Gauting, Germany) at magnification of ×85 200 (1.76 Å per pixel) and 1.0–2.8 μm defocus. A total of 16 micrographs were recorded for single particle analysis. All 16 micrographs were imported to EMAN2 (version 2.3) for further processing.^70^ After importing and estimating defocus with e2evalimage.py, single particles in different orientations were selected from the images using e2boxer.py in manual mode (11 094 particles, after one more manual selection, 10 223 particles were left). For each image, the contrast transfer function (CTF) parameters were estimated on boxed out regions (containing particles, 168 × 168) using e2ctf.auto.py program. A reference-free 2D classification based on the selected 10 223 phase-flipped particles (low-pass filtered to 20 Å) was performed using e2refine2d.py. The 2D classes show an approximate 2-fold symmetry, which is consistent with both the results of rh wildtype Bri2 BRICHOS oligomer and the biochemical data. Generated 2D classes were used as the input for building the 3D initial model using e2initialmodel.py. 3D refinement was performed in several rounds using e2refine_easy.py applying D2 symmetry aiming at a final resolution of 15 Å. The first two rounds of 3D refinements were performed with pixel size of 3.52 Å after binning the data by a linear factor 2. In the last round of refinement, the data was resampled to 2.464 Å per pixel. The final map from the first round of refinements was used as model in the second, and the final map from the second round of refinements was used as model in the third. The resolution was determined based on a Fourier shell correlation (FSC) value of 0.143,^71^ following the gold standard FSC procedure implemented in EMAN2.^72^

### BRICHOS and Aβ42 monomer interaction monitored by surface plasmon resonance

Aβ42 monomers were immobilized by amine coupling onto flowcell 4 on a CM5 sensor chip (GE Healthcare) using a BIAcore 3000 instrument (BIAcore AB). A reference surface was prepared on flowcell 3 using the same coupling protocol but without protein injected. The immobilization was performed at a flow rate of 20 μL min^−1^ with 20 mmol L^−1^ sodium phosphate pH 8.0 containing 0.2 mmol L^−1^ EDTA as running buffer, and the other details were set according to the manufacturer's instructions. After immobilization with a final reponse level of 727 RU, the flow-cells were stabilized over night in running buffer (10 mmol L^−1^ HEPES pH 7.5 containing 150 mmol L^−1^ NaCl and 0.2 mmol L^−1^ EDTA) at a flow rate of 20 μL min^−1^. For binding analysis, 25 μmol L^−1^ rh wildtype proSP-C BRICHOS or the D105N mutant in 10 mmol L^−1^ HEPES pH 7.5 containing 150 mmol L^−1^ NaCl and 0.2 mmol L^−1^ EDTA were injected in over the chip surfaces at 25 °C at a flow rate of 20 μL min^−1^ for 3 min, respectively. 10 mmol L^−1^ NaOH was used for further chip surface regeneration. For kinetic analysis, different concentrations of rh proSP-C BRICHOS D105N mutant in running buffer, *i.e.* 0, 1.56, 3.13, 6.25, 12.5, 25, 50 and 100 μmol L^−1^, were individually injected in over the chip surfaces at 25 °C at a flow rate of 20 μL min^−1^. 30 mmol L^−1^ NaOH was used for further chip surface regeneration. In all experiments, the response from the blank surface was subtracted from the immobilized surface response, the baselines of the sensorgrams were adjusted to zero and buffer spikes were excluded.

Steady state apparent affinities for rh proSP-C BRICHOS D105N mutant to immobilised Aβ42 monomers were estimated by plotting the maximum binding response *versus* BRICHOS concentrations. The baseline of the sensorgrams were adjusted to zero and buffer spikes were excluded for global fits to reflect the binding affinity. Since the response signals of the two lowest protein concentrations (*i.e.*, 1.56 and 3.13 μmol L^−1^) used in kinetic analysis were too weak, only sensorgrams obtained from rh proSP-C BRICHOS D105N mutant ranging from 6.25 μmol L^−1^ to 100 μmol L^−1^ were included in the global fits. The dissociation was globally fitted to a biexponential model as described by eqn (1):^24,73^

1 *R*(*t*) = *R*_1_(*x*e^−*k*_d1_(*t*−*t*_1_)^) + (1 − *x*)e^−*k*_d2_(*t*−*t*_1_)^

where *R*_1_ is the response signal at the starting time for the dissociation phase *t*_1_, and *x* is between 0 and 1. *k*_d1_ and *k*_d2_ are the dissociation rate constants for the fast and slow dissociation phases, respectively. The global fitted value *k*_d2_ was used to calculate the apparent *K*_D_ value.

The association phase was fitted to eqn (2):^24^

2 *R*(*t*) = *R*_f_ + (*R*_0_ − *R*_f_)e^−*k*_obs_*t*^

where *R*_0_ and *R*_f_ are the initial and final response signal of the association phase, respectively. *k*_obs_ is the observed rate constant described by eqn (3):^24,73^

3 *k*_obs_ = *ck*_a_ + *k*_d_

where *c* is the protein concentration, *k*_a_ is the association rate constant, and *k*_d_ from this analysis is related to secondary binding artifacts corresponding to *k*_d1_. Linear regression was used to determine *k*_a_. The apparent *K*_D_ value was calculated as ratio of the dissociation rate constant and association rate constant.

### Aβ42 monomer preparation and ThT assay

Recombinant Met-Aβ1–42 here referred to as Aβ42, was produced in BL21*(DE3) pLysS *E. coli* (B strain) cells and purified by ion exchange.^36^ The purified Aβ42 peptides were lyophilized and re-dissolved in 20 mmol L^−1^ Tris pH 8.0 with 7 mol L^−1^ Gdn-HCl, and the monomers were isolated in 20 mmol L^−1^ sodium phosphate pH 8.0 with 0.2 mmol L^−1^ EDTA by a Superdex 30 column 26/600 (GE Healthcare, UK). The concentration of monomeric Aβ42 was calculated with using an extinction coefficient of 1424 M^−1^ cm^−1^ for (*A*_280_–*A*_300_). For Aβ42 fibrillization kinetics analysis, 20 μL solution containing 10 μmol L^−1^ ThT, 3 μmol L^−1^ Aβ42 monomer and different concentrations of rh BRICHOS at molar ratios 0, 10, 30, 50, 70 or 100% (relative to Aβ42 monomer molar concentration), were added to each well of half-area 384-well microplates with clear bottom (Corning Glass 3766, USA), and incubated at 37 °C under quiescent conditions. The ThT fluorescence was recorded using a 440 nm excitation filter and a 480 nm emission filter using a microplate reader (FLUOStar Galaxy from BMG Labtech, Offenberg, Germany). For Aβ42 seeds preparation, 3 μmol L^−1^ Aβ42 monomers were incubated for about 20 h at 37 °C, and the fibrils were then sonicated in a water bath for 3 min. For seeding of Aβ42 fibrillization, 20 μL solution containing 10 μmol L^−1^ ThT, 3 μmol L^−1^ Aβ42, different concentrations of rh BRICHOS at 0, 10, 50, 70 and 100%, and 0.6 μmol L^−1^ seeds (calculated from the original Aβ42 monomer concentration) were added at 4 °C to each well in triplicate of 384-well microplates with clear bottom (Corning Glass 3766, USA) and immediately incubated at 37 °C under quiescent conditions. The elongation rate constant *k*_+_ in the presence of rh BRICHOS was calculated from the highly seeded experiments. The initial slope of the concave aggregation traces was determined by a linear fit of the first ∼20 min traces. For all the experiments, aggregation traces were normalized and averaged using four or three replicates, and data defining one dataset was recorded from the same plate at the same time, and all the ThT data was from the same batch of Aβ42 peptide.

### Analysis of Aβ42 aggregation kinetics

Fibrillization traces of Aβ42 with and without different concentrations of rh BRICHOS were fitted to a sigmoidal equation eqn (4),^30,36^ where the half time *τ*_1/2_ and the maximal growth rate *r*_max_ were extracted:

4 *F* = *F*_0_ + *A*/(1 + exp[*r*_max_(*τ*_1/2_ − *t*)])

where *A* the amplitude and *F*_0_ the base value.

To dissect the molecular mechanism underlying BRICHOS counteracting Aβ42 aggregation, the fibrillization traces were globally fitted by eqn (5):^23^5
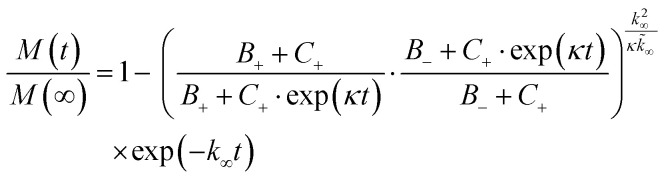
where *M*(*t*) is the total fibril mass concentration, and the intermediate coefficients are functions of *λ* and *κ*, and *n*_C_ and *n*_2_ are the reaction orders for primary and secondary nucleation, respectively:*C*_±_ = ±*λ*^2^/2/*κ*^2^
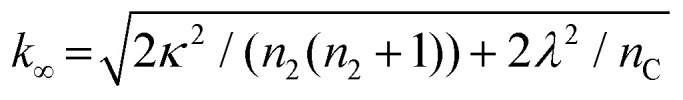

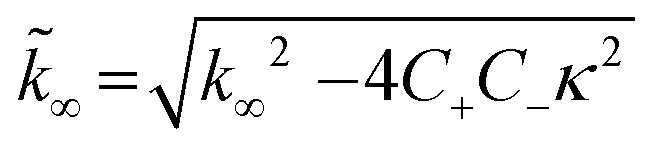
*B*_±_ = (*k*_∞_ ± *k̃*_∞_)/2/*κ*
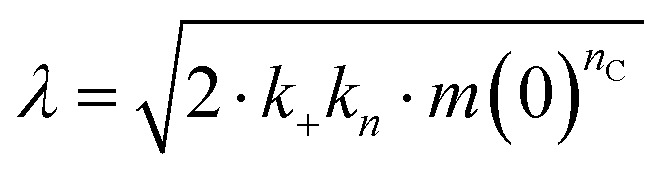

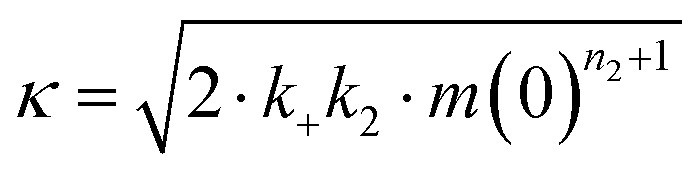


The microscopic rate constants *k*_*n*_, *k*_+_, and *k*_2_ are for primary nucleation, elongation, and secondary nucleation, respectively. The kinetic data were globally fitted to eqn (5), where the fits were partially constrained with one fitting parameter held to a constant value, resulting in that only one rate constant (*k*_*n*_, *k*_+_ or *k*_2_) is the sole fitting parameter.^30,36^ To investigate the generation of nucleation units, according to the nucleation rate *r*_*n*_(*t*):^23^*r*_*n*_(*t*) = *k*_*n*_*m*(*t*)^*n*_c_^ + *k*_2_*M*(*t*)*m*(*t*)^*n*_2_^,the total number of nucleation units was calculated by integrating the nucleation rate *r*_*n*_(*t*) over the reaction.

### Immunogold staining of Aβ42 fibrils and transmission electron microscopy analysis

Five μmol L^−1^ Aβ42 monomer was incubated at 37 °C with 100% rh wildtype proSP-C BRICHOS and the D105N mutant overnight, and the fibrils were collected at 4 °C by centrifugation for 1 h at 22 000 × *g*. The fibrils were gently resuspended in 20 μL 1 × TBS, of which 2 μL were applied to carbon coated copper grids, and incubated for about 5 min. Excess solution was removed and the girds were blocked by incubation in 1% BSA in 1 × TBS for 30 min, followed by 3 × 10 min washing by 1 × TBS. The grids were then incubated with polyclonal antibody against human proSP-C (SFTPC) (1 : 200 dilution, Atlas Antibodies) overnight at 4 °C, and washed 3 × 10 min with 1 × TBS. Finally, the grids were incubated with anti-rabbit IgG-gold coupled to 20 nm gold particles (1 : 40 dilution, BBI Solutions) for 2 h at room temperature, and washed 5 × 10 min with 1 × TBS. Excess solution was removed, and 2 μL of 2.5% uranyl acetate was added to each grid (kept about 20 s). Excess solution was removed, and the grids were air-dried at room temperature, and analysed by transmission electron microscopy (TEM, Jeol JEM2100F at 200 kV).

### Electrophysiological recordings

All the animal experiments were carried out in accordance with the ethical permit granted by Norra Stockholm's Djurförsöksetiska Nämnd (dnr N45/13). C57BL/6 mice of either sex (postnatal days 14–23, supplied from Charles River, Germany) were used in the experiments. Before sacrificed, all the mice were anaesthetized deeply using isoflurane.

The brain was dissected out and placed in modified ice-cold ACSF (artificial cerebrospinal fluid). The ACSF contained 80 mmol L^−1^ NaCl, 24 mmol L^−1^ NaHCO_3_, 25 mmol L^−1^ glucose, 1.25 mmol L^−1^ NaH_2_PO_4_, 1 mmol L^−1^ ascorbic acid, 3 mmol L^−1^ NaPyruvate, 2.5 mmol L^−1^ KCl, 4 mmol L^−1^ MgCl_2_, 0.5 mmol L^−1^ CaCl_2_ and 75 mmol L^−1^ sucrose. Horizontal sections (350 μm thick) of the ventral hippocampi from both hemispheres were sliced with a Leica VT1200S vibratome (Microsystems, Sweden). The sections were immediately transferred to a submerged incubation chamber containing standard ACSF: 124 mmol L^−1^ NaCl, 30 mmol L^−1^ NaHCO_3_, 10 mmol L^−1^ glucose, 1.25 mmol L^−1^ NaH_2_PO_4_, 3.5 mmol L^−1^ KCl, 1.5 mmol L^−1^ MgCl_2_ and 1.5 mmol L^−1^ CaCl_2_. The chamber was held at 34 °C for at least 20 min after dissection and it was subsequently cooled to room temperature (∼22 °C) for a minimum of 40 min. Proteins (Aβ42 and rh BRICHOS) were first added to the incubation solution for 15 min, and then the slices were transferred to the interface-style recording chamber for extracellular recordings. During the incubation, slices were supplied continuously with carbogen gas (5% CO_2_, 95% O_2_) bubbled into the ACSF.

Recordings were performed with borosilicate glass microelectrodes in hippocampal area CA3, pulled to a resistance of 3–5 MΩ, filled with ACSF and placed in stratum pyramidale. Local field potentials (LFP, *γ* oscillations) were recorded at 32 °C in an interface-type chamber (perfusion rate 4.5 mL per minute) and elicited by applying kainic acid (100 nmol L^−1^, Tocris). The oscillations were stabilized for 20 min before any recordings. No Aβ42, rh Bri2 BRICHOS R221E species or combinations thereof were present in the recording chamber either during *γ* oscillations stabilization, or during electrophysiological recordings. The interface chamber recording solution contained 124 mmol L^−1^ NaCl, 30 mmol L^−1^ NaHCO_3_, 10 mmol L^−1^ glucose, 1.25 mmol L^−1^ NaH_2_PO_4_, 3.5 mmol L^−1^ KCl, 1.5 mmol L^−1^ MgCl_2_ and 1.5 mmol L^−1^ CaCl_2_.

Interface chamber LFP recordings were carried out by a 4-channel amplifier/signal conditioner M102 amplifier (Electronics lab, University of Cologne, Germany). The signals were sampled at 10 kHz, conditioned using a Hum Bug 50 Hz noise eliminator (LFP signals only; Quest Scientific, North Vancouver, BC, Canada), software low-pass filtered at 1 kHz, digitized and stored using a Digidata 1322A and Clampex 9.6 software (Molecular Devices, CA, USA).

Power spectral density plots (from 60 s long LFP recordings) were calculated using Axograph X (Kagi, Berkeley, CA, USA) in averaged Fourier-segments of 8 192 points. Oscillation power was calculated from the integration of the power spectral density from 20 to 80 Hz.

### Statistics and reproducibility

The electrophysiological data is presented as means ± standard errors of the means. Prior statistical analysis all the data was subjected to outlier determination and removal with the ROUT method (*Q* = 1%) followed by D’Agostino & Pearson omnibus normality test. Based on the previous experience with the outliers and overall sample behaviour, sample size was determined based on previous studies performed in an interface-type chamber.^23,30,36,46,74,75^ Each experimental round was performed with parallel controls (Control KA and Aβ42) from the same animal and randomized preparations (slices incubated with Aβ42 + rh proSP-C BRICHOS D105N, + wildtype rh proSP-C BRICHOS, + rh Bri2-BRICHOS D148N monomers or + wildtype rh Bri2-BRICHOS). For comparison purposes data from Control KA and Aβ42 was pooled from interleaved slices recorded in these conditions. The number of recorded slices per condition (at least from 3–5 mice) are shown in the corresponding figure legend and source data file. Kruskal–Wallis test followed by Dunn's multiple comparisons were carried out due to the non-parametric nature- or relatively small size of some data. Comparison of the preventative efficacies of rh Bri2-BRICHOS D148N monomers and rh proSP-C BRICHOS D105N was assessed with the Mann Whitney test. Significance levels are * *p* < 0.05, ** *p* < 0.01, and ***p < 0.001. The ThT assay data are presented as means ± standard deviation, and the aggregation traces are averaged from four or three replicates.

## Data and materials availability

The density map of the Bri2 BRICHOS D148N oligomer have been deposited in the Electron Microscopy Data Bank (EMDB) under the accession code EMD-13005. All data and materials related to this paper are available from G. C. (gefei.chen@ki.se).

## Author contributions

G. C., Y. A. T., X. Z., H. P., H. B., A. L., and N. K. performed experiments. S. H. and G. C. carried out bioinformatic analyses and visualization. G. C., Y. A. T., X. Z., H. B., H. P., A. A., N. K., A. R., H. H., P. K., A. F., and J. J. analysed the data. G. C. and J. J. conceived the study. G. C. and J. J. wrote the paper. All authors discussed the results and commented on the manuscript.

## Conflicts of interest

The authors declare no competing financial interests.

## Supplementary Material

CB-003-D2CB00187J-s001

CB-003-D2CB00187J-s002
