# Supplementary material for: Abilities of the BRICHOS domain to prevent neurotoxicity and fibril formation are dependent on a highly conserved Asp residue
Source: RSC Chem Biol. 2022 Sep 15;3(11):1342–58. doi: 10.1039/d2cb00187j (PMC9627735; doi:10.1039/d2cb00187j)
Supplement: CB-003-D2CB00187J-s001 [file CB-003-D2CB00187J-s001.pdf]

Electronic Supplementary Material (ESI) for RSC Chemical Biology.
This journal is © The Royal Society of Chemistry 2022

Supplementary information

Ability of the BRICHOS domain to prevent neurotoxicity and fibril formation are dependent on a highly conserved Asp residue

Gefei Chen[1*], Yuniesky Andrade-Talavera[2#], Xueying Zhong[3#], Sameer Hassan[1#], Henrik Biverstal[1,4], Helen Poska[1,5], Axel Abelein[1], Axel Leppert[1], Nina Kronqvist[1], Anna Rising[1,6], Hans Hebert[3], Philip J.B. Koeck[3], André Fisahn[2], Jan Johansson[1]

[1]Department of Biosciences and Nutrition, Karolinska Institutet, 141 52 Huddinge, Sweden
[2]Department of Neurobiology, Care Sciences and Society, Center for Alzheimer Research, Division of Neurogeriatrics, Neuronal Oscillations Laboratory, Karolinska Institutet, 171 77 Stockholm, Sweden
[3]School of Engineering Sciences in Chemistry, Biotechnology and Health, Department of Biomedical Engineering and Health Systems, KTH Royal Institute of Technology, 141 52 Huddinge, Sweden
[4]Department of Physical Organic Chemistry, Latvian Institute of Organic Synthesis, Riga LV-1006, Latvia
[5]School of Natural Sciences and Health, Tallinn University, Tallinn, Estonia
[6]Department of Anatomy, Physiology and Biochemistry, Swedish University of Agricultural Sciences, 750 07 Uppsala, Sweden

# These authors contributed equally.
*Corresponding author, E-mail: gefei.chen@ki.se

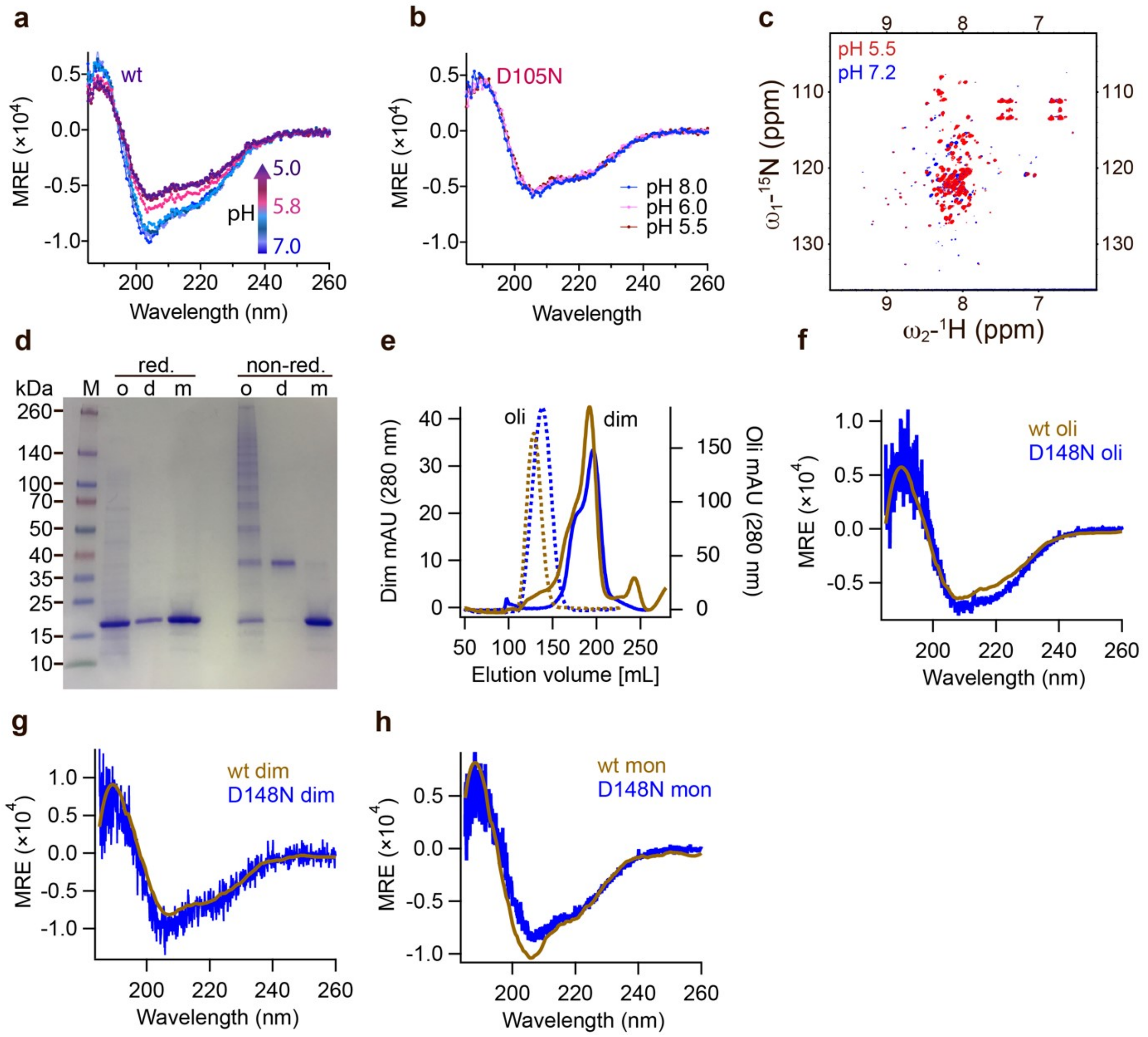

Fig. S1. Characterization of rh wildtype and Asp to Asn mutant BRICHOS. (**a** and **b**) CD measurements of rh wt proSP-C BRICHOS (purple) and the D105N mutant at different pHs. (**c**) NMR HSQC spectrum comparison of rh wt proSP-C BRICHOS (purple) and the D105N mutant at pH 7.2 (blue) and 5.5 (red). (**d**) SDS-PAGE analyses of SEC isolated rh Bri2 BRICHOS D148N species under reducing and non-reducing conditions. (**e**) SEC of wildtype (wt) Bri2 BRICHOS (blue) and the D148N (yellow) oligomer (dash line) and dimer (solid line) fractions prepared from corresponding fusion protein (NT*-Bri2 BRICHOS) oligomer and dimer. oli, oligomers; dim, dimers. (**f–h**) CD spectra of rh Bri2 BRICHOS D148N monomers, dimers and oligomers and the comparison to the corresponding rh wildtype (wt) species. MRE is the mean molar residual ellipticity in deg·cm^2·dmol^{-1}.

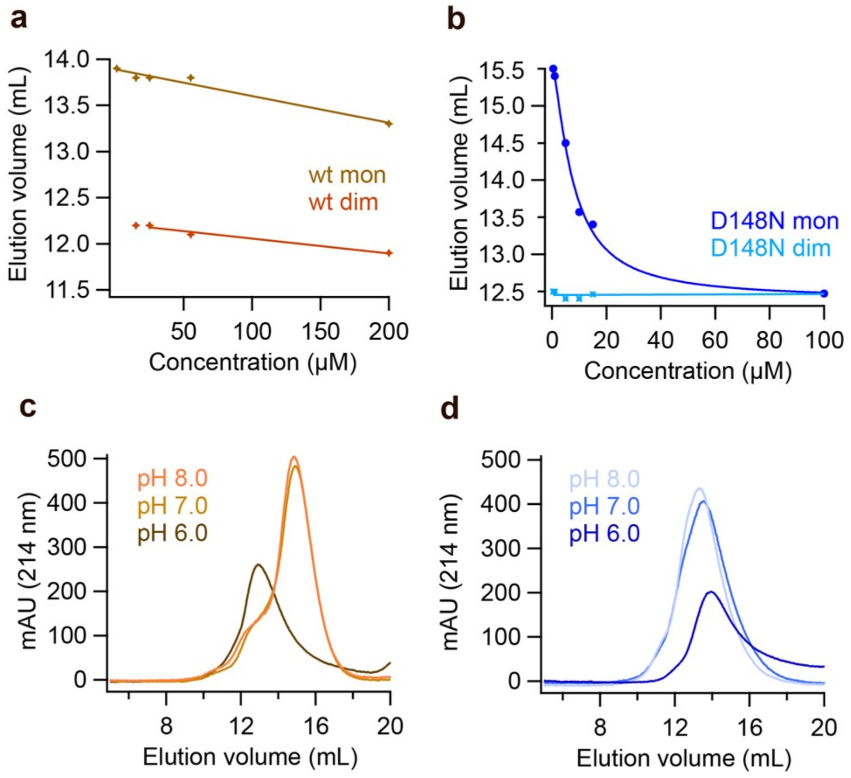

Fig. S2. Concentration and pH dependent properties of rh Bri2 BRICHOS. (**a**) Concentration-dependent dimerization of rh wildtype Bri2 BRICHOS monomers, the details are shown in **Supplementary Figure 3**. (**b**) Concentration-dependent dimerization of rh Bri2 BRICHOS D148N monomers, the details are shown in **Supplementary Figure 4**. pH dependent dimerization of (**c**) 10 µmol L^{-1} rh wildtype Bri2 BRICHOS monomers and (**d**) rh Bri2 BRICHOS D148N monomers analysed by SEC.

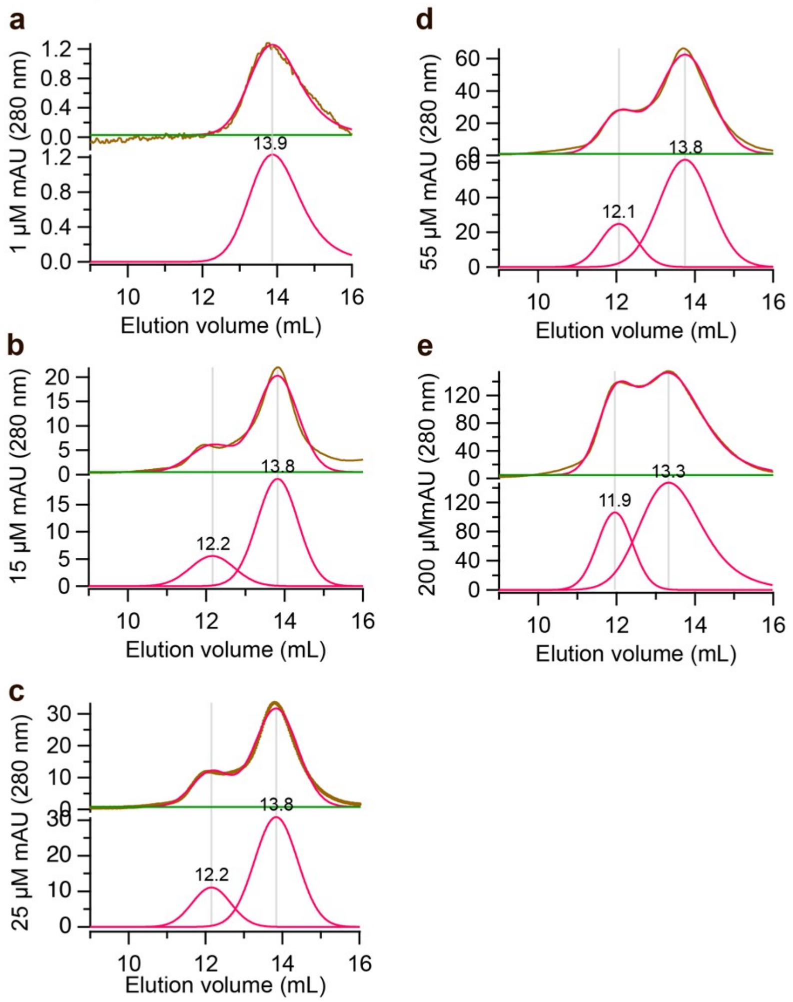

Fig. S3. Rh wildtype Bri2 BRICHOS monomer concentration-dependent dimerization. SEC analysis of rh wildtype Bri2 BRICHOS monomer at concentrations of 1 (**a**), 15 (**b**), 25 (**c**), 55 (**d**) and 200 µmol L^{-1} (**e**). The top panel of each sub-figure is the raw SEC data (yellow) with gaussian fitted (red). The lower panel of each sub-figure is the multiple gaussian peak fitting (red) and the number on the top of each peak represents the elution volume.

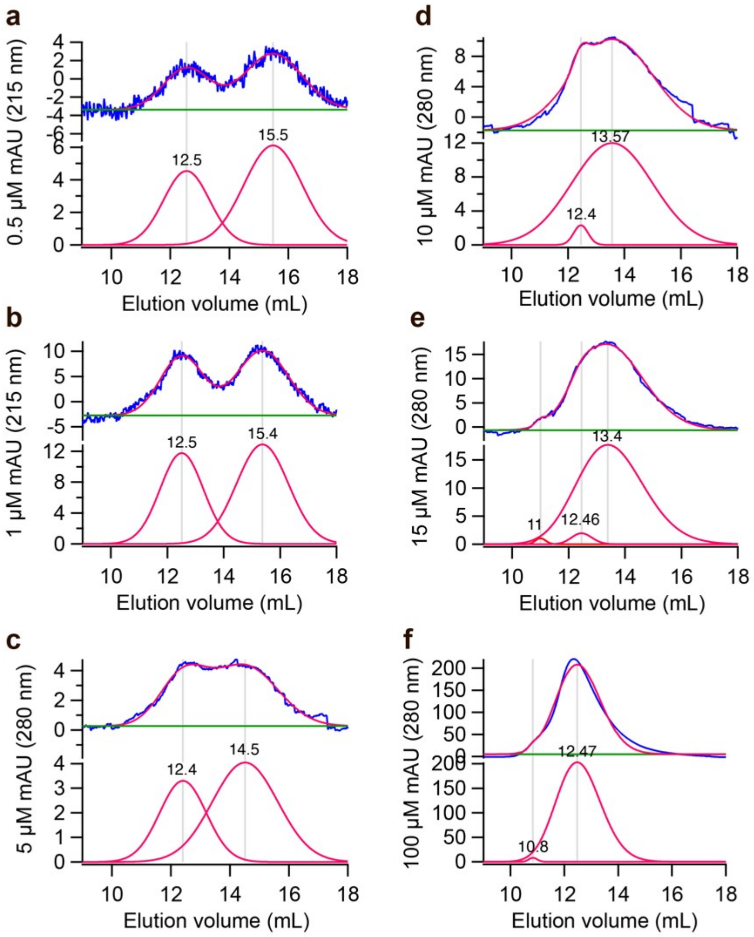

Fig. S4. Rh Bri2 BRICHOS D148N monomer concentration-dependent dimerization. SEC analysis of rh Bri2 BRICHOS D148N monomer at concentrations of 0.5 (**a**), 1 (**b**), 5 (**c**), 10 (**d**), 15(**e**) and 100 μmol L^{-1} (**f**). The top panel of each sub-figure is the unprocessed data (yellow) with gaussian fitted (red). The lower panel of each sub-figure is the gaussian peak fitting (red) and the number on the top of each is the relevant elution volume.

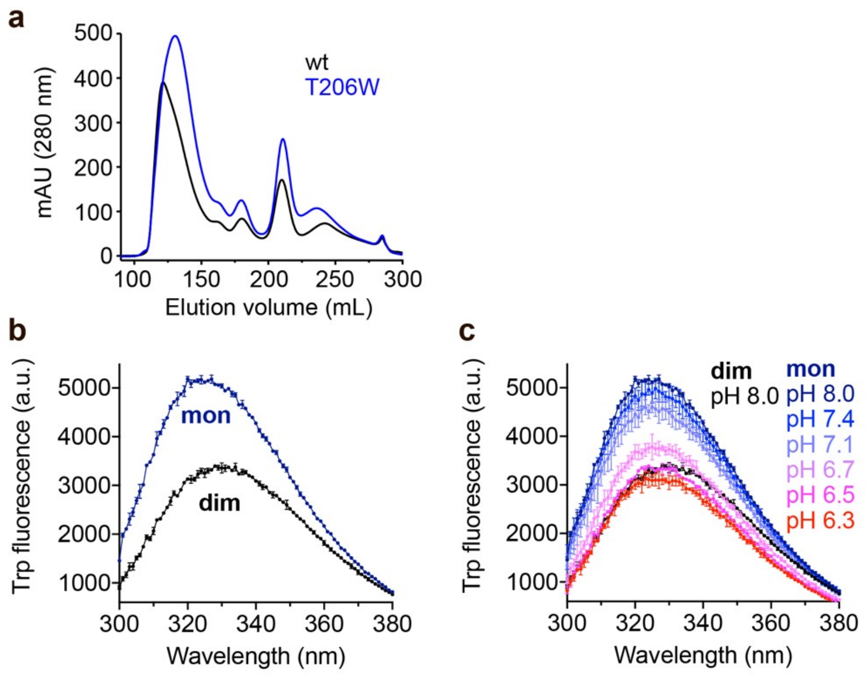

Fig. S5. Trp fluorescence of rh Bri2 BRICHOS T206W mutant. (**a**) SEC of rh wildtype NT*-Bri2 BRICHOS and the rh NT*-Bri2 BRICHOS T206W mutant. (**b**) Rh Bri2 BRICHOS T206W monomers and dimers were diluted to 2 µmol L^{-1} in 20 mmol L^{-1} NaPi, 0.2 mmol L^{-1} EDTA at pH 8.0. (**b**) Rh Bri2 BRICHOS T206W monomers at different pHs from pH 6.3 to pH 8.0 were subjected to Trp fluorescence measurements, while the dimers were measured at pH 8.0. All the samples were prepared in duplicates, and excited at 280 nm (5 µm bandwidth) and fluorescence emission from 300–400 nm (10 µm bandwidth,1 nm step interval) was recorded. For the final fluorescence intensities, the results were corrected by subtracting the background fluorescence. The data are presented as means ± standard deviations.

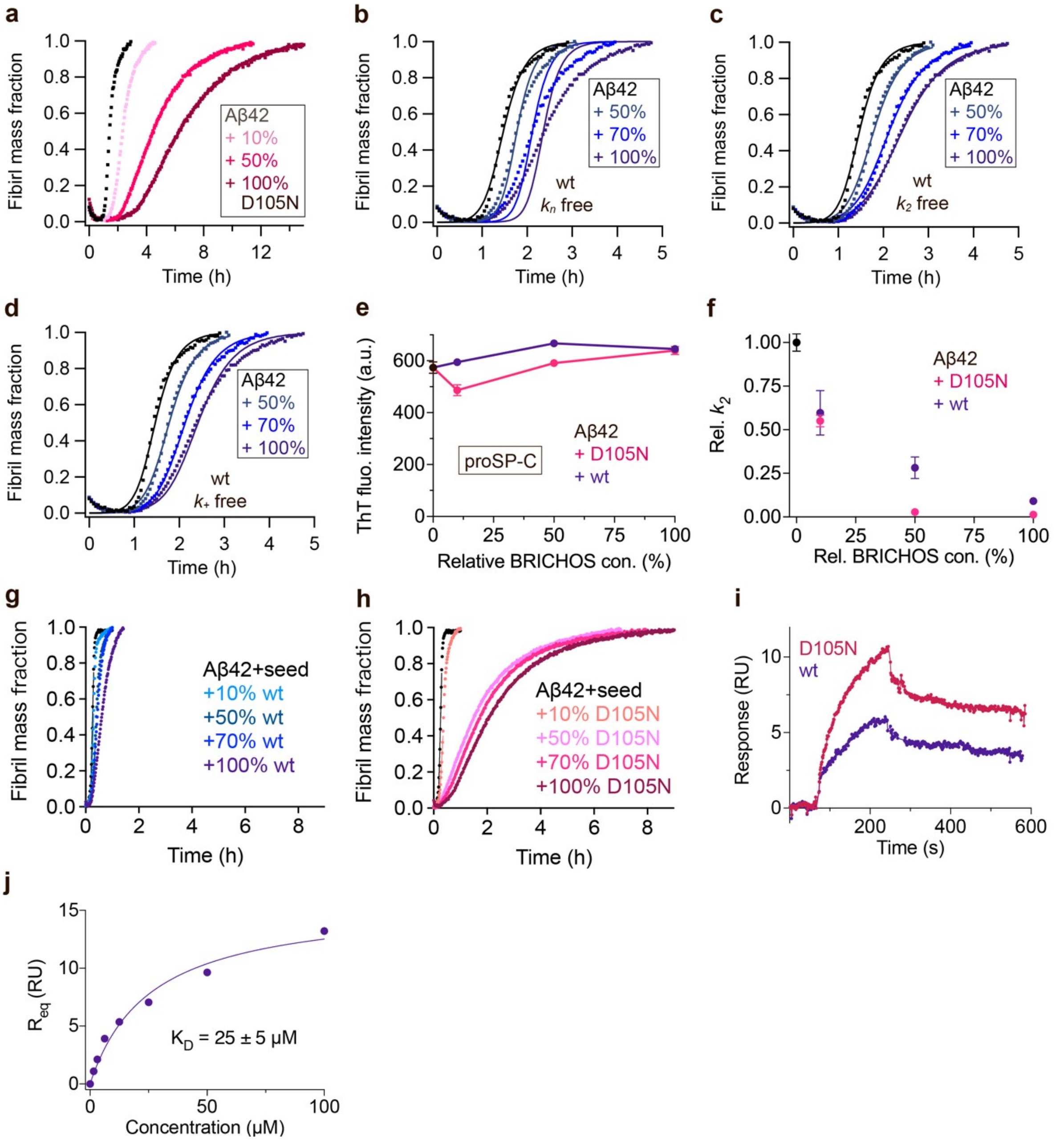

Fig. S6. Rh wildtype proSP-C BRICHOS interferes with Aβ42 fibril formation. (**a**) Aggregation kinetics of 3 μmol L^{-1} Aβ42 in the presence of rh proSP-C BRICHOS D105N at concentrations: 0 (black), 10 (light red), 50 (red), or 100% (dark red) molar percentage referred to monomeric subunits relative to Aβ42 monomer. (**b–d**) Aggregation kinetics of 3 μmol L^{-1} Aβ42 in the presence of rh wildtype (wt) proSP-C BRICHOS at concentrations: 0 (black), 50 (cyan), 70 (blue), or 100% (purple) molar percentage referred to monomeric subunits relative to Aβ42 monomer concentration. The global fits (solid lines) of the aggregation traces (cross) were constrained such that only one single rate constant is the free fitting parameter. (**e**) Final ThT fluorescence intensity of 3 μM Ab42 in the presence of rh proSP-C BRICHOS variants at

different concentrations. (**f**) Relative secondary nucleation rate constant k_2 of 3 μM Ab42 in the presence of rh proSP-C BRICHOS variants at different concentrations.

(**g** and **h**) Seeded aggregation traces of 3 μmol L^{-1} Aβ42 with 0.6 μmol L^{-1} preformed Aβ42 fibril seeds in the presence of different concentration of rh wildtype proSP-C BRICHOS (**f**) and the D105N mutant (**h**). (**i**) Analysis of rh wildtype (wt) proSP-C BRICHOS (purple) and the D105N mutant (red) binding to Aβ42 monomers detected by SPR. 25 μmol L^{-1} rh wildtype proSP-C BRICHOS and the D105N mutant were injected in over the chip surfaces, respectively. The response from the blank surface was subtracted from the immobilized surface response. (**j**) Steady state affinity for rh proSP-C BRICHOS D105N to immobilised Aβ42 monomers. Different concentrations of rh proSP-C BRICHOS D105N mutant, *i.e.* 0, 1.56, 3.13, 6.25, 12.5, 25, 50 and 100 μmol L^{-1}, were individually injected over the chip surfaces. Steady state affinity was estimated by plotting the maximum binding responses versus BRICHOS concentrations.

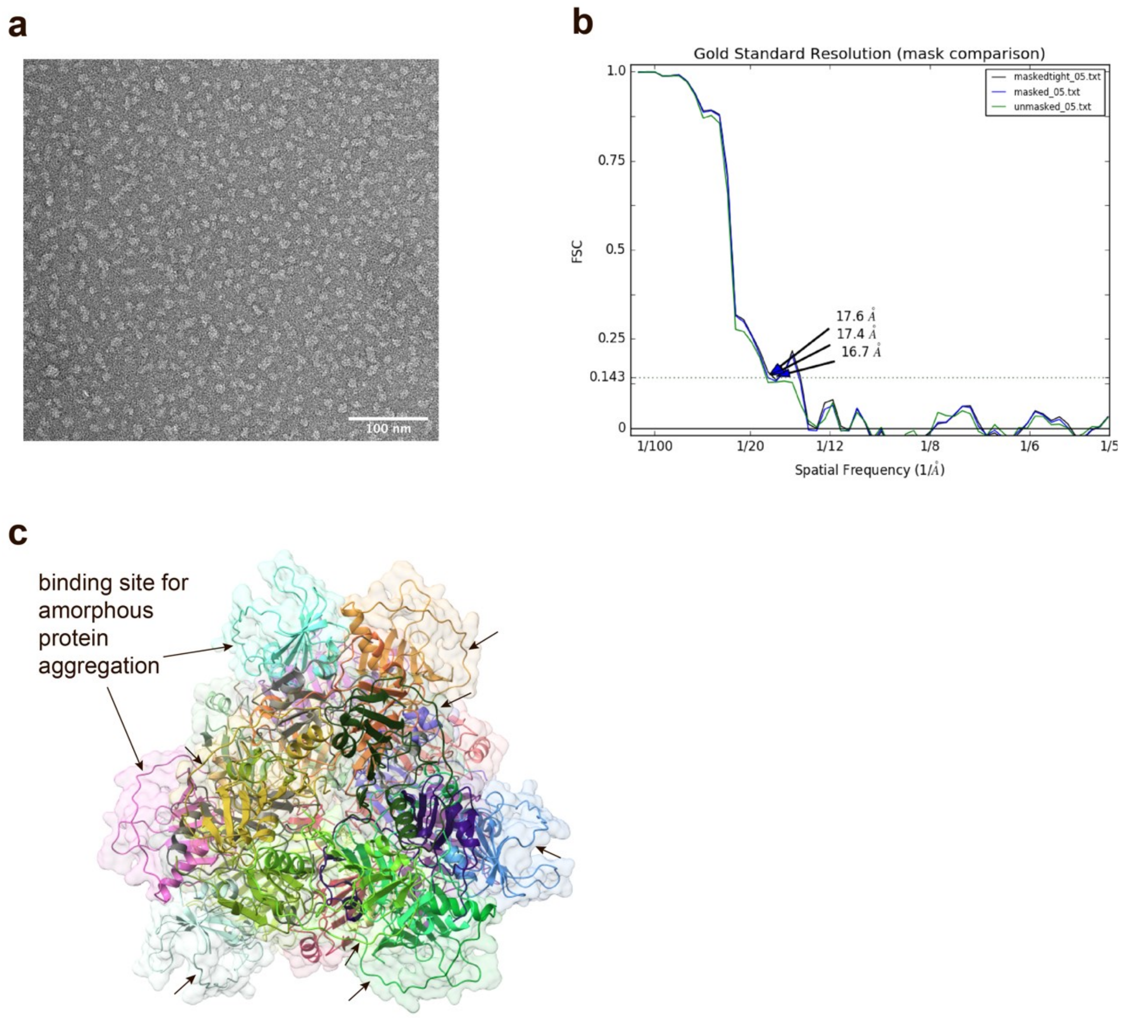

Fig. S7. Electron microscopy analyses of rh Bri2 BRICHOS D148N oligomers. (**a**) Raw TEM micrograph of negatively stained rh Bri2 BRICHOS D148N oligomers. (**b**) The Fourier shell correlation (FSC) indicates a resolution of ~17 Å for the reconstructed 3D density map determined at an FSC-value of 0.143 (dotted lines). (**c**) The solvent exposed loops of the rh Bri2 BRICHOS oligomers model responsible for general chaperone activity against amorphous aggregation are indicated by arrows.

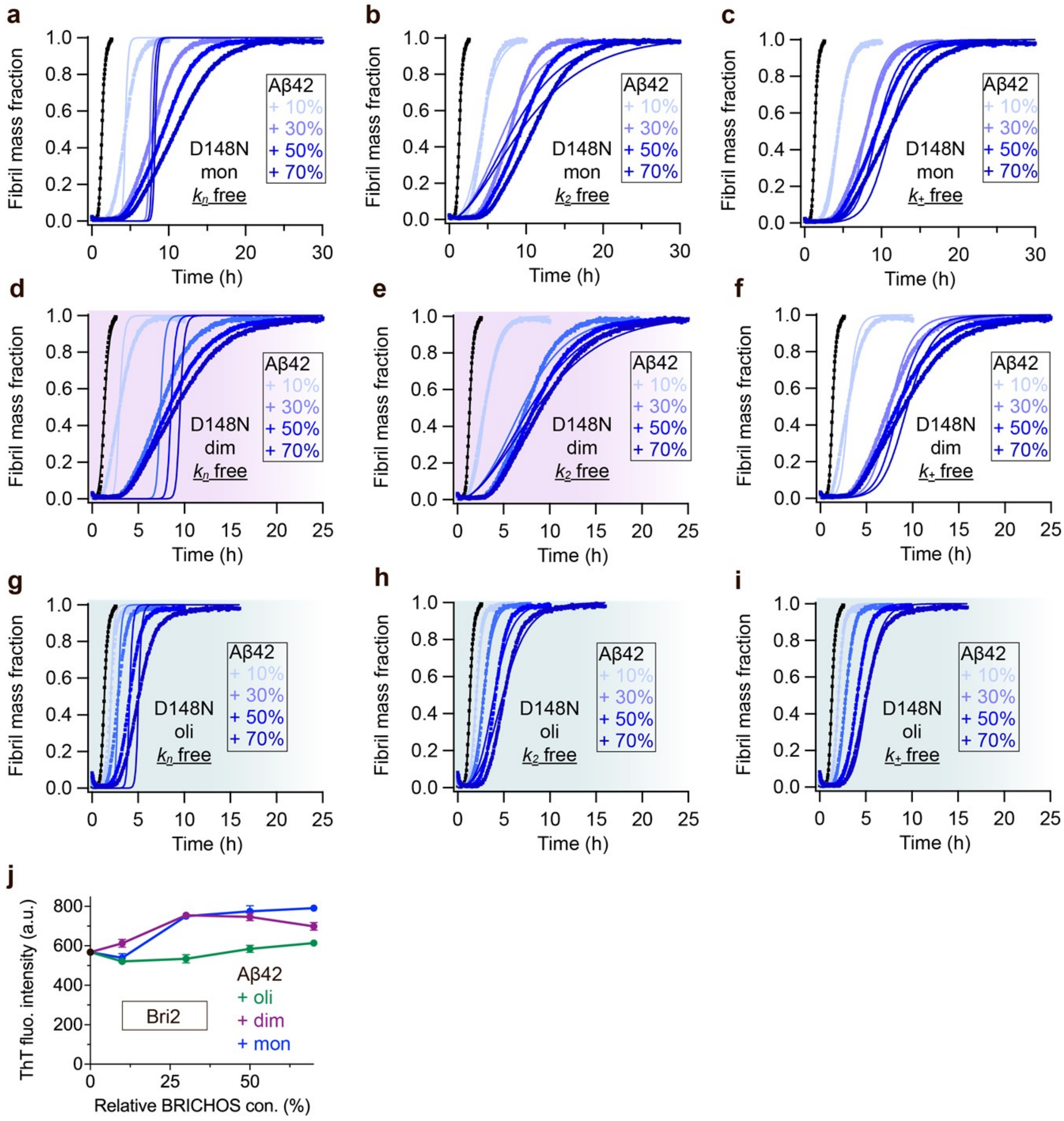

Fig. S8. Rh Bri2 BRICHOS D148N inhibition of Aβ42 fibril formation. (**a–c**) Aggregation kinetics of 3 µmol L^{-1} Aβ42 in the presence of rh Bri2 BRICHOS D148N monomers at different concentrations. The global fits (solid lines) of the aggregation traces (dot) were constrained such that only one single rate constant is the free fitting parameter, indicated in each panel. χ^2 values describing the quality of the fits: 62 for k_n free, 11.2 for k_2 free and 3.7 for k_+ free. (**d–f**) Aggregation kinetics of 3 µmol L^{-1} Aβ42 in the presence of rh Bri2 BRICHOS D148N dimers at different concentrations. The global fits (solid lines) of the aggregation traces (dot) were constrained such that only one single rate constant is the free fitting parameter, indicated in each panel. χ^2 values describing the quality of the fits: 43 for k_n free, 3.9 for k_2 free and 7.8 for k_+ free. (**g–i**) Aggregation kinetics of 3 µmol L^{-1} Aβ42 in the presence of rh Bri2 BRICHOS D148N oligomers at different concentrations. The global fits (solid lines) of the

aggregation traces (dot) were constrained such that only one single rate constant is the free fitting parameter. χ^2 values describing the quality of the fits: 8.6 for k_n free, 1.4 for k_2 free and 0.4 for k_+ free. For the different D148N species, both k_2 and k_+ as sole free fitting rate, the fibrillization traces were described with similar χ^2 values, suggesting both k_2 and k_+ might be affected, like the wildtype species. (j) Final ThT fluorescence intensity of 3 μM Ab42 in the presence of rh Bri2 BRICHOS variants at different concentrations.

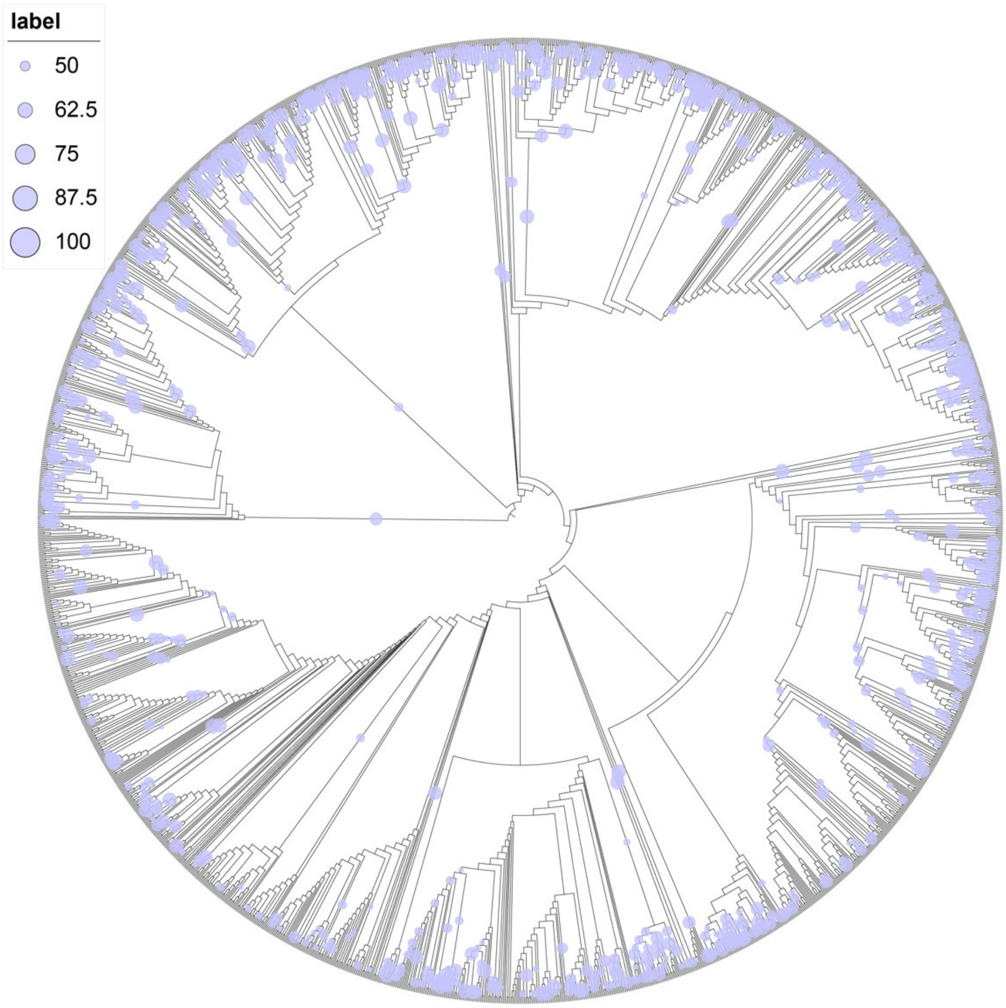

Fig. S9. Evolution of the molecular chaperone domain BRICHOS. Phylogenetic tree of the 2 019 BRICHOS domains, which was grouped into thirteen families (**Fig. 6c**). The bootstraps larger than 50% are indicated by labels, where the sizes are proportional to the bootstrap values.

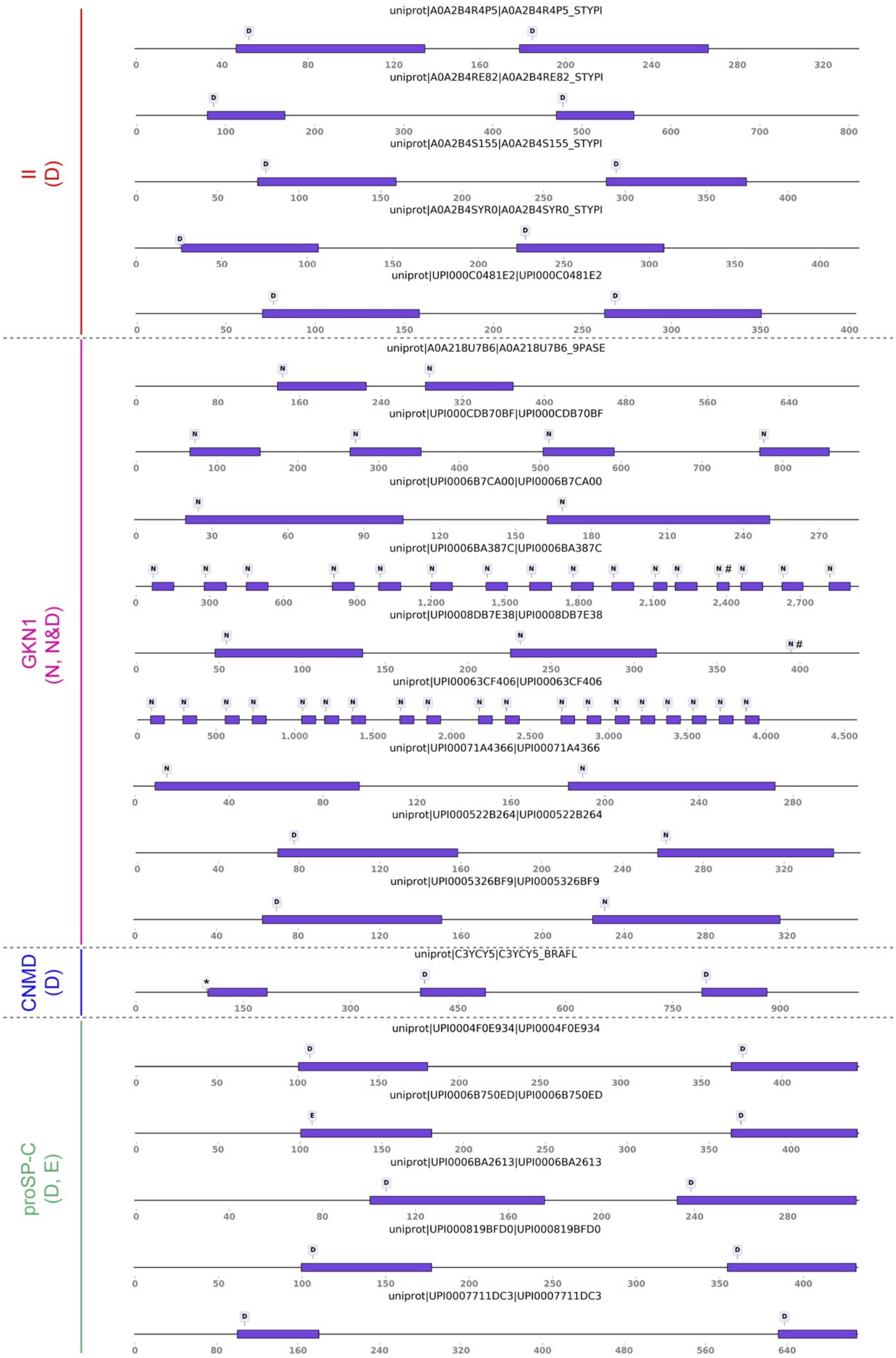

Fig. S10. The architecture of proproteins containing multiple BRICHOS domains. The proproteins contain up to nineteen multiple, tandem BRICHOS domains (multi-BRICHOS). The architecture is generally shown in Figure 1d. The uniport accession number is shown over

each corresponding cartoon. The length of the bar is not proportional to the size of the BRICHOS domain from different precursors. # refers to the C-terminally truncated, and * indicated the N-terminally truncated.

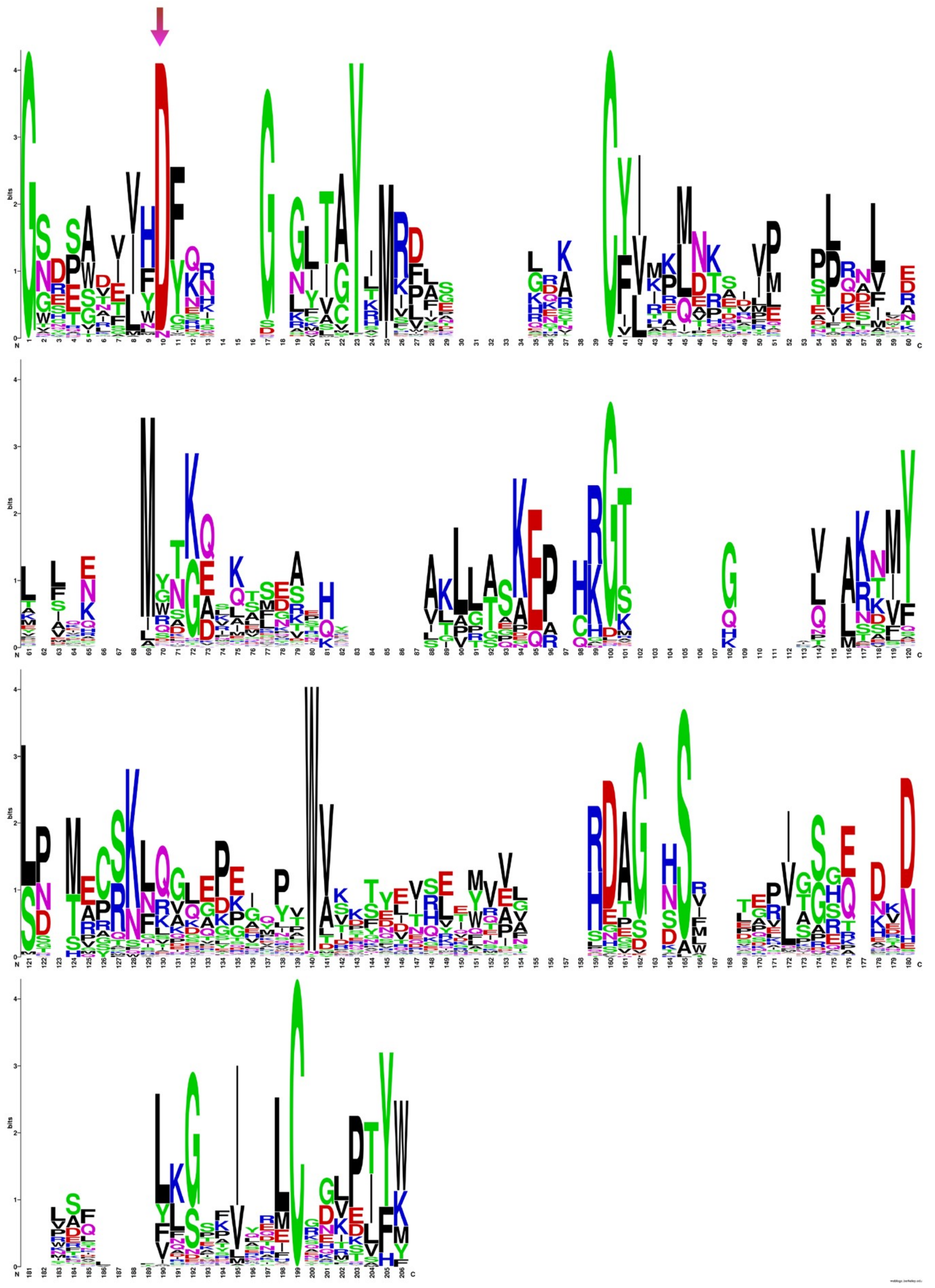

Fig. S11. WebLogo representation of the 1 908 single BRICHOS domains. The height of the residue stack implies the sequence conservation, while the height of symbols within the

stack indicates the relative frequency of each residue. The arrow marks the conserved Asp residue.

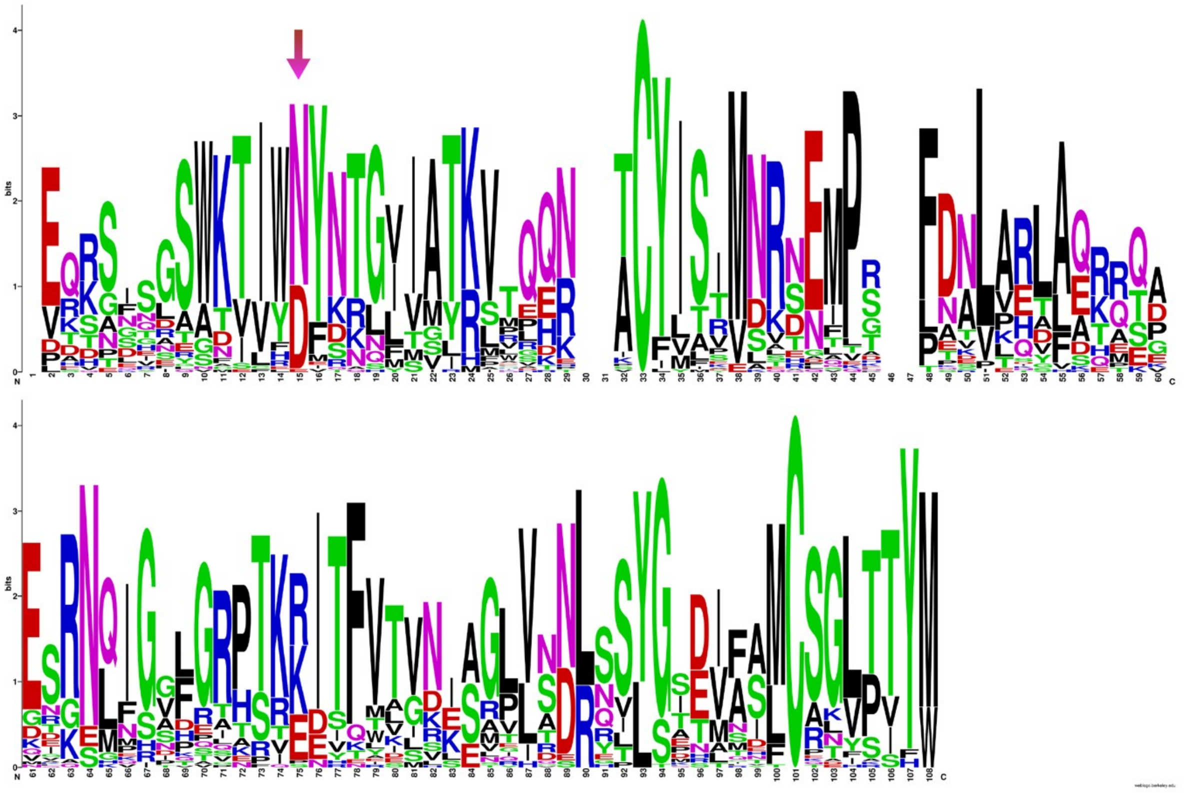

Fig. S12. WebLogo representation of the 74 multiple BRICHOS domains. The height of the residue stack implies the sequence conservation, while the height of symbols within the stack indicates the relative frequency of each residue. The arrow marks the conserved Asp residue.

Supplementary Table.

Table S1. Species distribution and pairwise alignment of the multiple BRICHOS domains. See separate file.
